# The Evolution of Seabirds in the Humboldt Current: New Clues from the Pliocene of Central Chile

**DOI:** 10.1371/journal.pone.0090043

**Published:** 2014-03-12

**Authors:** Martín Chávez Hoffmeister, Jorge D. Carrillo Briceño, Sven N. Nielsen

**Affiliations:** 1 School of Earth Sciences, University of Bristol, Bristol, United Kingdom; 2 Palaeontological Institute and Museum, University of Zürich, Zürich, Switzerland; 3 Instituto de Ciencias Ambientales y Evolutivas, Universidad Austral de Chile, Valdivia, Chile; Team ‘Evo-Devo of Vertebrate Dentition’, France

## Abstract

**Background:**

During the last decade, new Neogene fossil assemblages from South America have revealed important clues about the evolution of seabird faunas in one of the major upwelling systems of the world: the Humboldt Current. However, most of this record comes from arid Northern Chile and Southern Peru and, in consequence, our knowledge of the evolutionary history of seabirds in the temperate transitional zone is negligible. A new Late Pliocene assemblage of fossil birds from the coastal locality of Horcon in Central Chile offers a unique opportunity to fill this gap.

**Principal Findings:**

Isolated bones of a medium-sized penguin are the most abundant bird remains. Morphological and cladistic analyses reveal that these specimens represent a new species of crested penguin, *Eudyptes calauina* sp. nov. *Eudyptes* is a penguin genus that inhabit temperate and subantarctic regions and currently absent in central Chile. Additionally, a partial skeleton of a small species of cormorant and a partial tarsometatarsus of a sooty shearwater have been identified.

**Conclusion/Significance:**

The Horcon fossils suggest the existence of a mixed avifauna in central Chile during the Pliocene in concordance with the latitudinal thermal gradient. This resembles the current assemblages from the transitional zone, with the presence of species shared with Northern Chile and Southern Peru and a previously unrecorded penguin currently absent from the Humboldt System but present in the Magellanic region. Comparison of Pliocene seabird diversity across the Pacific coast of South America shows that the Horcon avifauna represents a distinctive assemblage linking the living faunas with the Late Miocene ones. A comparison with the fossil record near the Benguela Current (west coast of southern Africa) suggests that the thermic gradient could play an important role in the preservation of a higher diversity of cold/temperate seabirds in the Humboldt Current.

## Introduction

The fossil record of seabirds is often abundant in Cenozoic marine formations. Nevertheless, there is a clear asymmetry in our knowledge of fossil seabird communities between the Northern and Southern hemispheres. Of the 368 records of seabirds presented by Warheit [1], only 25% come from the Southern Hemisphere and of those, 27% come from South America. Fortunately, the number of taxa reported for South America had been increasing in abundance and diversity during the last decade, including two of the most important Neogene assemblages: the Late Miocene Bahia Inglesa Formation in northern Chile [2]–[4], and the Middle Miocene to Pliocene Pisco Formation in Southern Peru [5]–[7]. These are key areas for the study of the evolution of seabird faunas in one of the major upwelling systems of the world: the Humboldt Current.

The Humboldt Current system [8] is one of the most productive marine ecosystems worldwide and its area of influence defines one of the largest biogeographical provinces in the southern oceans: the Peruvian-Chilean Province (PCP) [9], which has been recognized as one of the main areas of endemism for seabirds in Chile [10]. Some authors also recognize a transition zone at its southern limit, between 30 and 43°S, also known as the Central [11] or Central Chilean Province [12], where this fauna becomes progressively more similar to the Magellanic fauna by the addition of cold-temperate taxa [13].

Unfortunately, the Neogene record of seabirds in other marine formations of the Southeast Pacific is comparatively scarce and consequently poorly known [14]–[18]. Most of these records are Pliocene in age, being younger than the main assemblages recorded in Bahia Inglesa and Pisco, and giving us an exceptional opportunity for the study of changes in the composition of seabird faunas over time. However, all these localities are restricted to the PCP in Northern Chile and Southern Peru. In consequence, our knowledge of the evolutionary history of seabirds in the transitional zone during the Neogene is negligible.

In this context, the discovery of a new assemblage of fossil birds in the coastal locality of Horcon in central Chile offers a unique opportunity to fill this gap for southern temperate areas and, along with the previously known Pliocene records, reveals changes in the composition of seabird faunas in the area of influence of the Humboldt Current during the last 5 Ma.

## Materials and Methods

### The Horcon Formation

The specimens described here come from coastal outcrops of the Horcon Formation [19], [20], located 51 km north to the city of Valparaiso between the villages Horcon and Maitencillo in the Valparaiso Region, central Chile (see Figure S1.1 in Document S1).

The lithology of the Horcon Formation is characterized by predominance of subhorizontal, unconsolidated fine sandstones with a dip of 2° to 3°. The estimated thickness of the sequence is 45 m and two well-defined stratigraphic intervals can be identified. All the specimens described here come from the upper unit, which corresponds to the main section of the sequence (see Figure S1.2 in Document S1). This unit is characterized by layers of fine to coarse sandstone, light-colored and poorly consolidated, which are interspersed with few conglomeritic layers. Vertebrate and invertebrate macrofossils are abundant in all the sandstone strata, with over 60 taxa recognized so far, making the Horcon Formation one of the most diverse and the southernmost marine vertebrate assemblages currently known for the Neogene in the Southeastern Pacific [21]. The stratigraphic column for this formation can be found in Document S1.

Tavera [20] assigned the Horcon Formation to the Pliocene based on the mollusk biostratigraphy. The new mollusk specimens collected during this study, which include the bivalves *Chlamys* cf. *hupeanus* and *Panopea coquimbensis* along with the gastropods *Chorus blainvillei*, *Chorus doliaris* and *Herminespina mirabilis*, corroborate an age not younger than Late Pliocene [22]–[28].

### Repository information

The material consists of eighteen specimens including a set of associated wings and pectoral girdle elements (SGO-PV 21443) deposited in the Vertebrate Palaeontological collection of the Museo Nacional de Historia Natural, Santiago (Chile), under accession numbers SGO-PV 21443 to 21455 and SGO-PV 21487 to 21490. All necessary permits were obtained for the described study, which complied with all relevant regulations. The collection of these specimens was authorized by the Consejo de Monumentos Nacionales (Chile) through the order number 4703, enacted on September 24th, 2010.

### Nomenclatural Acts

The electronic edition of this article conforms to the requirements of the amended International Code of Zoological Nomenclature, and hence the new names contained herein are available under that Code from the electronic edition of this article. This published work and the nomenclatural acts it contains have been registered in ZooBank, the online registration system for the ICZN. The ZooBank LSIDs (Life Science Identifiers) can be resolved and the associated information viewed through any standard web browser by appending the LSID to the prefix “http://zoobank.org/”. The LSID for this publication is: urn:lsid:zoobank.org:pub: 5DE13597-E734-453B-8703-F42F84f206A38. The electronic edition of this work was published in a journal with an ISSN, and has been archived and is available from the following digital repositories: PubMed Central, LOCKSS.

### Phylogenetic analysis

**Figure 1:**
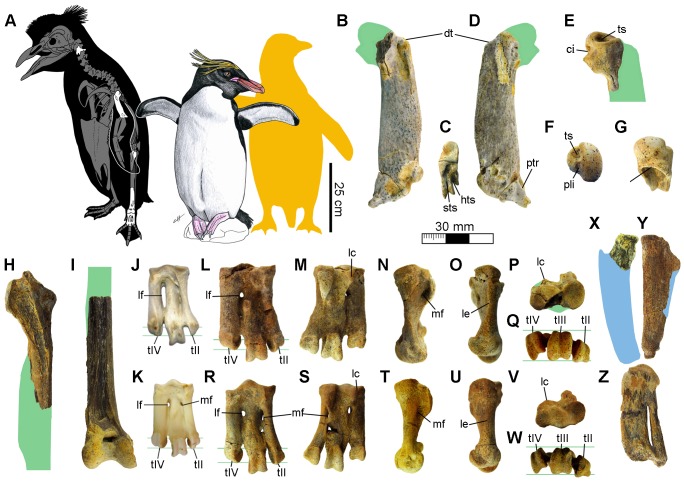
Fossil penguins from the Horcon Formation. **A.** Skeletal reconstruction of *Eudyptes calauina* sp. nov. showing the identified elements; and size comparison with the Macaroni penguin *E. chrysolophus* and the Yellow-eyed penguin *Megadyptes antipodes* (yellow silhouette). Referred specimens: left humerus (SGO-PV 21451) in (**B**) cranial, (**C**) distal and (**D**) caudal views; proximal fragment of left humerus (SGO-PV 21449) in (**E**) cranial, (**F**) proximal and (**G**) ventral views; (**H**) proximal fragment of right tibiotarsus (SGO-PV 21447) in cranial view; and (**I**) distal portion of right tibiotarsus (SGO-PV 21488) in cranial view. Tarsometatarsus of Humboldt penguin *Spheniscus humboldti* (**J**) and Northern Rockhopper penguin *E. moseleyi* (**K**). Holotype: right tarsometatarsus attributed to an adult (SGO-PV 21487) in (**L**) dorsal, (**M**) plantar, (**N**) medial, (**O**) lateral, (**P**) proximal and (**Q**) distal views. Paratype: right tarsometatarsus attributed to a subadult (SGO-PV 21444) in (**R**) dorsal, (**S**) plantar, (**T**) medial, (**U**) lateral, (**V**) proximal and (**W**) distal views. Spheniscidae indet.: (**X**) proximal fragment of right radius (SGO-PV 21450) in ventral view; (**Y**) right ulna (SGO-PV 21455) in ventral view; and (**Z**) right carpometacarpus (SGO-PV 21454) in ventral view. Silhouettes (green for *E. calauina*, blue for Spheniscidae indet.) based on complementary specimens attributed to the same species or living relatives. **Abbreviations:** ci, capital incisure; dt, dorsal tubercle; hts, humerotricipital sulcus; lc, lateral hypotarsal crest; le, lateral edge of the metatarsi IV; lf, lateral proximal vascular foramen; mf, medial proximal vascular foramen; pli, pit for ligament insertion; ptr, posterior trochlear ridge; sts, scapulotricipital sulcus; tf, tricipital fossa; tII, trochlea metatarsi II; tIII, trochlea metatarsi III; tIV, trochlea metatarsi IV; ts, transverse sulcus.

To explore the phylogenetic relationship of the new penguin species described here (Figure 1), we expanded and modified a recently published combined matrix [29], including 246 morphological characters plus five genes (RAG-1, 12S, 16S, COI, and cytochrome b) with over 6000 basepairs. We added eight new osteological characters for the humerus and tarsometatarsus, new states for five of the previously used characters and a modification of the definition of two other characters. The list of characters, detail of the modifications to the original matrix and the GenBank accession numbers are provided in Document S1; and a nexus file of the entire data set is provided as Dataset S1.

Three of the taxa previously included by Ksepka *et al.* [29] were identified as wildcards, labile taxa that reduce the resolution of the consensus tree, and excluded from the final analysis: *Delphinornis wimani*, *Palaeeudyptes antarcticus* and *Duntroonornis parvus*. From the taxa added by Ksepka and Thomas [30], only *Inguza predemersus* was included in the final analysis; *Nucleornis* was included during preliminary analysis, but was identified as a wildcard and excluded from the final analysis. As a result, the current analysis includes 55 penguin taxa. All South American taxa included here were coded by direct observation, with the only exception of “*Pygoscelis*” *grandis*. The outgroup includes 13 species of Procellariiformes and two species of Gaviiformes. The trees were rooted on Gaviiformes.

The phylogenetic analysis was conducted following the strategy defined by Ksepka *et al.* [29], using PAUP4.0b10 [31] with a heuristic search strategy (1000 replicates of random taxon addition saving 10 trees per replicate, with TBR branch swapping). All characters were equally weighted, multistate coding was used only to represent polymorphism, and branches with a minimum length of zero were collapsed. A morphology-only and morphology plus molecular data analysis were done. Strict and Adams consensus trees were calculated for each analysis, but only the strict consensus are presented, showing the best-solved topology. Additional consensus trees can be found in the Figure S3 in Document S1.

## Results

### Systematic palaeontology

Sphenisciformes Sharpe, 1891.

Spheniscidae Bonaparte, 1831.

*Eudyptes* Vieillot, 1816.

**Figure 2:**
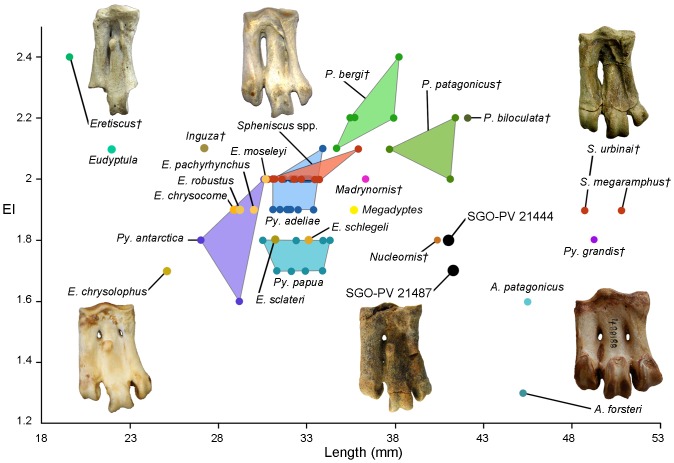
Tarsometatarsus elongation index vs length. Plot of the tarsometatarsus elongation index (EI, obtained from the division of the proximodistal length per the mediolateral width at the proximal end) against the proximodistal length of tarsometatarsus in Neogene penguins.

*Eudyptes calauina* sp. nov.

(Figures 1A–I, L–W, 2, Figure S2A–B in Document S1)

#### ZooBank life science identifer (LSID) for species

urn:lsid:zoobank.org:act: ECFC692D-4AA8-4AE6-8B56-307B416A432F

#### Etymology

Noun in apposition. Calauina (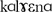) is the name of the rockhopper penguin (*Eudyptes chrysocome*) in the quasi-extinct Yaghan language, spoken by the Yagán people from Tierra del Fuego, Southern Chile.

#### Holotype

SGO-PV 21487, complete right tarsometatarsus (Figure 1L–Q, Figure S2A in Document S1).

#### Paratype

SGO-PV 21444, complete right tarsometatarsus (Figure 1R–W, Figure S2B in Document S1).

#### Referred materials

SGO-PV 21452, cervical vertebrae; SGO-PV 21451, left humerus lacking humeral head (Figure 1B–D); SGO-PV 21448, distal portion of left humerus; SGO-PV 21449, proximal fragment of left humerus (Figure 1E–G); SGO-PV 21447, proximal portion of right tibiotarsus (Figure 1H); SGO-PV 21488, distal portion of right tibiotarsus (Figure 1I); SGO-PV 21445, pedal phalanx; SGO-PV 21453, fragment of pedal phalanx.

#### Type locality and horizon

Horcon Formation, Late Pliocene, Chile. SGO-PV 21487, 21452, 21448, 21449, 21488 and 21453 collected from layer 12 (Figure S1.2 in Document S1). SGO-PV 21444 collected from layer 8. SGO-PV 21451 collected from layer 9. SGO-PV 21445 collected from layer 10.

#### Diagnosis

Larger than *Megadyptes antipodes*, *Spheniscus chilensis* and *S. humboldti*; but smaller than *S. urbinai* and *Pygoscelis grandis*. The tarsometatarsus is on average 30% larger than *Eudyptes sclateri*, 16% larger than *Megadyptes antipodes* and 12% larger than *Madrynornis*. Based on the humerus, *E. calauina* can be differentiated from other species of the genus by (i) the presence of a slightly concave and asymmetrical proximal border of the tricipital fossa in ventral view instead of the stronger symmetrical concavity common in extant species, (ii) a more robust humeral shaft with a robustness index (proximodistal length/ventrodorsal width at middle point) of 4 whereas in extant species it is between 4.5 and 4.9 (see Character 176 in Document S1), and (iii) a scapulotricipital sulcus separated from the humerotricipital sulcus and not dorsally connected as in other species. At the level of the tarsometatarsus, it can be differentiated by (i) an elongation index between 1.7 and 1.8 (Figure 2), (ii) a moderately deep medial dorsal sulcus instead of the occasionally shallow one observed in other species, (iii) a strongly dorsoplantarly compressed lateral edge of metatarsus IV creating a sharp edge in lateral view, unlike the wider and rounded edge in extant species, and (iv) a slightly pointed trochlea II with parallel medial and lateral edges in plantar view, instead of the strongly pointed one with a more rounded medial edge of extant species. An expanded description of the specimens assigned to *E. calauina* is provided in Document S1.

#### Measurements

SGO-PV 21487: length at middle point 41.3 mm; proximal mediolateral width 24.3 mm; distal mediolateral width 27.5 mm. SGO-PV 21444: length at middle point 41 mm; proximal mediolateral width 22.7 mm; distal mediolateral width 25.3 mm. SGO-PV 21451: maximum preserved length 76.8 mm; diaphysis length 69.4 mm; ventrodorsal width at middle point 17.4 mm. A comparative table of measurements for the tarsometatarsus is provided in Table S1 in Document S1.

### Anatomical remarks

Although all specimens have been found isolated, all of them are similar in morphology to homologous elements of the genus *Eudyptes* and larger than in *Megadyptes*. The most diagnostic elements of this set are the tarsometatarsus (SGO-PV 21487 and SGO-PV 21444) and humerus (SGO-PV 21451 and SGO-PV 21449); both elements widely used for the typification of fossil penguins and showing the same distinctive combination of characters as in *Eudyptes*, whereas all the other specimens are referred mainly based on their size range and general morphology. Consequently, only the tarsometatarsus and humerus are used for the diagnosis.

This new species can be assigned to the genus *Eudyptes* based on a combination of 23 osteological characters. Of these, ten allow us to discriminate this species from *Spheniscus* (Figure 1J), the only penguin genus that currently inhabits the area and the most common one in the fossil record of Chile and Peru. These characters are, at level of humerus: (i) humeral head strongly prominent proximally (slightly prominent in *Eudyptula* and *Spheniscus*); (ii) notch between dorsal tubercle and humeral head present (usually absent in *Spheniscus*, *Eudyptula* and *Tereingaornis*); (iii) capital incisure completely separated from transverse ligament sulcus (connected through narrow sulcus in *Aptenodytes*, *Inguza*, *Madrynornis*, *Palaeospheniscus* and *Eretiscus*); (iv) presence of deep pit for ligament insertion adjacent to head on proximal surface (absent or very shallow in *Pygoscelis antarctica*, *P. adeliae*, *Madrynornis*, *Palaeospheniscus*, *Eretiscus* and occasionally in *Aptenodytes patagonicus*); (v) proximal margin of tricipital fossa weakly projected in proximal view (well-exposed in extant species of *Spheniscus* and occasionally in *Pygoscelis antarctica*); (vi) impressio insertii m. supracoracoideus and m. latissimus dorsi separated by small gap (separated by moderate gap in *Palaeospheniscus*, *Eretiscus* and occasionally in *Spheniscus urbinai*); (vii) shaft robustness index between 4 and 5 (between 5 and 6 in *Eudyptula*, *Inguza*, *Madrynornis* and *Eretiscus*); (viii) nutrient foramen situated on ventral face of shaft (situated on anterior face in *Madrynornis* and *Eretiscus*); (ix) preaxial angle weak or absent (well defined in *Aptenodytes*, *Pygoscelis*, *Megadyptes*, *Spheniscus*, *Palaeospheniscus*, *Eretiscus* and occasionally in *Eudyptes*); (x) posterior trochlear ridge reaching ventral edge of the shaft (extends beyond ventral margin in *Aptenodytes*, *Pygoscelis* and *Madrynornis*; but does not reach ventral edge in *Inguza*, *Eretiscus* and some species of *Spheniscus*); (xi) trochlear angle greater than or equal to 45° (between 35° and 45° in *Inguza*, *Madrynornis*, *Tereingaornis* and occasionally slightly under 45° in *Spheniscus urbinai*, *Palaeospheniscus* and *Eretiscus*); and (xii) ulnar condyle almost parallel to radial and not surpassing anterior edge of humerus (slightly surpassing anterior edge in *Madrynornis*, *Palaeospheniscus* and *Eretiscus*).

At level of tarsometatarsus: (xiii) elongation index less than 2 (between 2 and 2.5 in extant species of *Spheniscus*, *S. muizoni*, *Megadyptes*, *Eudyptula*, *Inguza*, *Madrynornis*, *Palaeospheniscus*, *Eretiscus* and occasionally in *Pygoscelis adeliae* and *Eudyptes moseleyi*); (xiv) inconspicuous colateral lateral ligament scar (creating depression over lateral surface in *Pygoscelis*; and creating notch on proximolateral vertex in *Spheniscus*, *Eudyptula*, *Inguza*, *Nucleornis*, *Madrynornis*, *Palaeospheniscus* and *Eretiscus*); (xv) medial hypotarsal crest projected farther than lateral crest (both reach same projection in *Pygoscelis*); (xvi) intermediate hypotarsal crest indistinguishable from lateral crest (slightly separated by shallow groove in proximal view in *Madrynornis*, *Palaeospheniscus*, *Eretiscus* and occasionally in *Eudyptula* and *Eudyptes chrysocome*); (xvii) lateral hypotarsal crest forming diagonal ridge that overhangs lateral foramen (poorly defined and proximal to lateral foramen in *Aptenodytes*, *Pygoscelis*, *Megadyptes*, *Eudyptula*, *Spheniscus* and *Nucleornis*), (xviii) large medial proximal vascular foramen opening plantarly at medial surface of medial hypotarsal crest (often smaller in *Spheniscus* and *Eudyptula*; opening at plantar surface in *Aptenodytes*, *Pygoscelis* and *Nucleornis*; vestigial in *Palaeospheniscus* and *Eretiscus*); (xix) small lateral proximal vascular foramen (occasionally enlarged in *Spheniscus*; vestigial in *Eretiscus*; and absent in *Nucleornis*); (xx) lateral intertrochlear notch deeper than medial (sub-equal to equal in *Aptenodytes*, *Pygoscelis*, *Megadyptes*, *Spheniscus urbinai*, *S. megaramphus* and *Inguza*); (xxi) trochlea metatarsi IV shorter than II in dorsal view (sub-equal to equal in *Aptenodytes*, *Pygoscelis*, *Megadyptes*, *Eudyptula* and *Spheniscus*); (xxii) trochlea metatarsi III and IV aligned at the same plane in distal view (trochlea IV displaced dorsally in extant species of *Spheniscus* and *S. megaramphus*); and (xxiii) trochlea metatarsi II slightly deflected plantarly in distal view (strongly deflected in *Eudyptula*, *Spheniscus megaramphus* and *Palaeospheniscus*).

The tarsometatarsus SGO-PV 21487 (Figure 1L–Q) is 16% larger than in *Megadyptes*; whereas the best-preserved humerus available SGO-PV 21451 (Figure 1B–D) is approximately 5% larger than in *Megadyptes*. The elongation index (EI), obtained from the division of the proximodistal length per the mediolateral width at the proximal end of the tarsometatarsus, is 1.7 for SGO-PV 21487 and 1.8 for SGO-PV 21444 being within the range of *Eudyptes chrysolophus* (1.7), *E. sclateri* (1.8), *E. schlegeli* (1.8), *Pygoscelis papua* (1.7–1.8), *P. antarctica* (1.6–2.0), *P. grandis* (1.8) and *Nucleornis* (1.8). This is smaller than in *E. moseleyi*, *E. chrysocome*, *E. pachyrhynchus* and *E. robustus*. In a plot of the EI against the proximodistal length (Figure 2), it is evident that both specimens are separated from South American extant and fossil penguins; with *Nucleornis* from the Early Pliocene of South Africa being the only similar taxon in size and proportions. However, the EI of *Nucleornis* is based on an approximation of the proximal width and the morphology of this element is clearly distinguishable from *E. calauina*.

### Ontogenetic stages of specimens

Taking as reference the extant species of *Eudyptes* and *Megadyptes*, where the average length of the tarsometatarsus is approximately 45% of the length of the humerus, SGO-PV 21451 is approximately 13% smaller than the size expected based on the holotype (91.7 mm). We estimate the expected size range for the humerus, calculated as the linear measure +/−1.96 per the standard deviation [32], using the standard deviation for the humerus length in *E. pachyrhynchus* (2.1) [33]. The expected size range is between 87.6 and 95.8 mm, suggesting that SGO-PV 21451 is approximately 10% smaller than expected based on the proportions of living species of *Eudyptes*. Considering the relatively smooth surface texture of the humerus SGO-PV 21451, along with the well-defined edges and muscular attachments, this specimen can be attributed to an adult or late subadult [34]. In consequence, the difference in proportion observed cannot be easily attributed to the aging of the individuals. This can be an indication of a slightly different humerus-tarsometatarsus proportion in *E. calauina* as has been described in fossil species of *Spheniscus* [35]. There is evidence of variation in the average proportion represented by the tarsometatarsus among *Eudyptes* species: 45.8% in *E. chrysocome*, 45.4% in *E. pachyrhynchus*, 45.3% in *E. robustus* and 42% in *E. sclateri*. Considering a reconstructed length of 80 mm for the humerus SGO-PV 21451, and assuming that it belongs to the same individual as the holotype, the proportion in *E. calauina* will be close to 50%; being 5% higher than in most of the living species. This is similar to the proportion in *Eudyptula* (50%) and similar to the range of difference between *Aptenodytes patagonicus* (40.2%) and *A. forsteri* (34%). Nevertheless, it is clear that both elements belong to different individuals and the lack of associated elements does not allow a more detailed comparison.

On the other hand, it is important to note that whereas the holotype SGO-PV 21487 can be attributed to an adult (Figure 1L–Q), the paratype SGO-PV 21444 most likely represents a subadult individual (Figure 1R–W). This is based on the slight size difference, the more porous texture of the bone (Figure S2.A–B in Document S1) and the degree of development of some anatomical features in SGO-PV 21444, like the larger medial foramen and the deeper and more angular intertrochlear notches. For this reason, the diagnosis for the tarsometatarsus is based mostly on the holotype. Nevertheless, both specimens share the most diagnostic characters of the genus.

### Phylogenetic analysis

**Figure 3:**
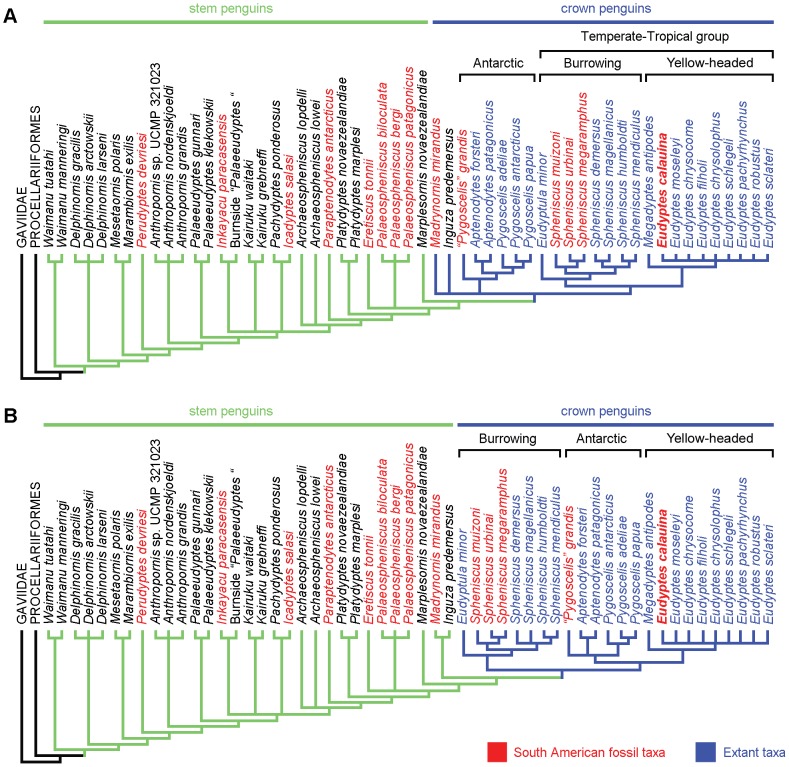
Phylogenetic relations of *Eudyptes calauina* sp. nov. **A.** Strict consensus tree of 192 MPTs (tree length = 5563 steps, rescalated consistency index [RC] = 0.373, retention index [RI] = 0.699) from a combined analysis of morphological characters plus >6000 bp. **B.** Strict consensus tree of 704 MPTs (tree length = 802 steps, RC = 0.492, RI = 0.879) from an analysis of morphological-only characters.

Analysis of the combined data set resulted in 192 most parsimonious trees (MPTs) of 5563 steps (Figures 3A, Figure S3.1-2 in Document S1), whereas the morphology-only analysis resulted in 704 MPTs of 802 steps (Figure 3B, Figure S3.3-4 in Document S1). Both analyses recovered fewer trees than Ksepka *et al.* [29], a difference attributed primarily to the exclusion of the wildcard taxa and a more complete data set. Our results also show better resolution and recover all the genera as monophyletic, with the exception of *Archaeospheniscus* and *Pygoscelis*. Topologies of the combined and morphology-only strict consensus trees are almost identical for the stem penguin taxa, but there is disagreement in the topology of the crown group between both analyses (Figure 3).

The general relations among the stem genera are similar to those previously reported [29], [30]; however, a new series of pairings has been obtained. *Mesetaornis* and *Marambiornis* are joined close to the base of the Sphenisciformes. The large polytomy recovered by Ksepka *et al.* [29], including *Palaeeudyptes*, *Inkayacu*, *Icadyptes* and *Pachydyptes* is better resolved. The two Antarctic species attributed to *Palaeeudyptes*, *P. gunnari* and *P. klekowskii*, are now separated from this polytomy as a more basal node. Three clades, composed of *Inkayacu*+the Burnside “*Palaeeudyptes*”, *Kairuku waitaki+Kairuku grebneffi* and *Icadyptes*+*Pachydyptes* are also recovered. They appear in a polytomy in both strict consensus trees (Figure 3). Additionally, *Palaeospheniscus* is recovered as monophyletic with respect to *Eretiscus*. A list of the osteological synapomorphies that support these clades and the monophyly of the extant genera can be found in Document S1.

Our results show a better-resolved topology for the crown group than previous analyses at level of genera [29], [30]. All the fossil taxa recovered as part of the crown group, with the possible exception of *Inguza*, are from the Neogene of South America. The strict consensus of our combined analysis shows *Madrynornis* and *Inguza* in an unsolved relationship with the clade containing the Antarctic penguins (*Aptenodytes*+*Pygoscelis*) and the temperate-tropical group containing the remaining extant genera (Figure 3A); whereas the morphological analysis joins both genera as the sister group of the crown Spheniscidae (Figure 3B). These taxa are also recovered outside Spheniscidae in the Adams consensus of the combined analysis (Figure S3.2 in Document S1). *Madrynornis* from the Middle Miocene of Argentina had been previously recovered as sister of *Eudyptes* within Spheniscidae [30], [36]; while in the case of *Inguza* from the Early Pliocene of South Africa, a closer relationship with the temperate-tropical group had been previously suggested [30]. This change is mainly the result of the modification of some characters and the substantial decrease in the percentage of missing-data for *Madrynornis*, which is reduced from 47.7 to 15.5 for osteological characters. In contrast with the most recent analyses [29], [30], the inclusion of both taxa within Spheniscidae slightly reduces the resolution of the strict consensus of the combined analysis in comparison with the morphology-only analysis. This decrease in resolution can be linked to a general problem regarding osteological characters: the high intrageneric homogeneity and intraspecific variation observed among extant taxa. In consequence, some of these characters must be coded as polymorphic, reducing their strength in comparison with the molecular data and collapsing some nodes in the combined analysis. It is expected that the addition of new characters and the reduction of missing data for fossil taxa will improve the resolution of the analyses as will improving completeness of molecular data for extant taxa.

All our analyses recover three main clades within Spheniscidae: (i) the Antarctic penguins, (ii) the burrowing penguins, and (iii) the yellow-headed penguins (Figure 2). The first one includes the truly Antarctic penguins joining the great penguins (*Aptenodytes*) and brush-tailed penguins (*Pygoscelis*). This clade has been previously recovered by morphology-only analyses [37], [38] and more recently by a molecular analysis [39], but both genera are always recovered in separate basal nodes in most molecular [40] and combined analyses [29], [30], [41]. In our results, the Antarctic group also includes the fossil species “*Pygoscelis*” *grandis* joined in a basal node. “*Pygoscelis*” *grandis* was originally included as a stem taxon of *Pygoscelis* [37], but subsequent analyses recovered it in different positions within the crown group [29], [41]. Unfortunately, the character states for this taxon have been taken from the literature in the latest analyses, so that a direct revision of the type specimen is required to resolve their affinities.

The burrowing penguins include the blue penguin (*Eudyptula*) and the banded penguins (*Spheniscus*), and had been recovered as a monophyletic group by molecular, morphological, and combined analysis [29], [39], [40], [41]. Within the banded penguins, a dichotomy between the extant species and the South American fossils is always recovered in our analysis. This differs from the relations presented by previous studies [29], in which *S. muizoni* is more closely related to the crown *Spheniscus* than to *S. urbinai* and *S. megaramphus*. This change is mainly due to the increase in the number of characters coded for the South American fossils.

Finally, the yellow-eyed penguin (*Megadyptes*) plus the crested penguins (*Eudyptes*) form the yellow-headed penguins clade, well supported by molecular, morphological, and combined analysis [29], [39], [40], [41]. *Eudyptes calauina* is always recovered within the crested penguins in our analysis. Despite the lack of internal resolution in the strict consensus (Figure 3), the crested penguins are always recovered as a monophyletic clade with the yellow-eyed penguin as sister taxon. The lack of internal structure in the strict consensus within *Eudyptes* is most likely related to the high percentage of missing molecular data, which in some species exceeds 80%. Nevertheless, part of the internal topology is recovered by the Adams consensus (Figure S3.2, S3.4 in Document S1)

The main difference between the combined and morphology-only analysis is the relationship between these three main clades derived from the rooting [42]. A temperate-tropical group (clade A in [30]) containing the burrowing and yellow-headed penguins has always been recovered as monophyletic in molecular [39], [40] and combined analyses [29], [30], [41] (Figure 3A), and the Adam consensus of our combined analysis recovers a dichotomy between the Antarctic penguins and the temperate-tropical penguins (Figure S3.2 in Document S1). On the other hand, morphology-only analyses often recover the burrowing penguins as the most basal node within Spheniscidae, joining the Antarctic and yellow-headed penguins in a more derived clade [37], [38], [41] (Figure 3B). This topology is collapsed in some of the most recent morphological trees [29], [30], most likely due to the inclusion of wildcard taxa. It is important to mention, that this does not affect the placement of *Eudyptes calauina* within the crested penguins; and despite these differences, the main relations between genera are largely congruent. It has been suggested that the study of fossil taxa representing the proximal outgroups to the crown Spheniscidae could help to improve its rooting [42]. In this sense, our results suggest that the study of the relationships of *Madrynornis* and *Inguza* can be key to improve the congruence between molecular and morphological data.

### Spheniscidae indet

#### Referred materials

SGO-PV 21489, fragment of right coracoid; SGO-PV 21450, proximal fragment of right radius (Figure 1X); SGO-PV 21455, right ulna (Figure 1Y); SGO-PV 21454, right carpometacarpus (Figure 1Z); SGO-PV 21457, distal fragment of left carpometacarpus. All specimens collected from layer 12. Measurements in table 1.

**Table 1:** Length measured and expected for flipper element.

	Measured	Specimen measured	Expected	Percentage of total length
Humerus	76.8^a^ (80^b^)	SGO-PV 21451	70.1+/−4.5	33%
Ulna	53.1	SGO-PV 21455	51	24%
Carpometacarpus	42.5	SGO-PV 21454	42.5	20%

The expected lengths were calculated base on the proportions described for *Eudyptes*, *Spheniscus* and *Megadyptes* [42], and using the carpometacarpus SGO-PV 21454 as reference specimen. The range for the humerus was calculated based in the method of Warheit [32] (measure +/− 1.96 x standard deviation) and using the standard deviation offered by Livezey [42] for the humerus of *Spheniscus magellanicus* (2.3). Note that the humerus SGO-PV 21451 is larger than the expected length.

a . Maximum conserved length,

b . Estimated total length.

### Remarks

All the specimens included here are morphologically similar to *Spheniscus* and *Eudyptes* and in the size range of *Spheniscus humboldti* and *S. chilensis*, being approximately 20% smaller than the size expected for *E. calauina*. These may represent a second and smaller species of penguin or juveniles of *E. calauina*.

The fibrous textures observed in the ulna SGO-PV 21455 (Figure 1Y, Figure S2.C in Document S1) and the carpometacarpus SGO-PV 21454 (Figure 1Z) allow us to attribute these specimens to immature individuals. Based on the proportions of the flipper elements in *Eudyptes*, *Spheniscus* and *Megadyptes* [43], and the length of the carpometacarpus SGO-PV 21454, we calculate the expected length for the main elements of the appendicular skeleton (Table 1). The recorded length of the ulna SGO-PV 21455 is congruent with the expected size. However, the humerus SGO-PV 21451 is approximately 12% larger than the expected length based on the carpometacarpus. These differences suggest that both specimens belong to two separate taxa or, as the surface texture suggests, to different ontogenetic stages, whereas the ulna and carpometacarpus possibly belong to the same taxon and a similar ontogenetic stage. Unfortunately, the fragmentary nature of these specimens and the fact that at least some of them belong to immature individuals, make it impossible to offer a more specific assignation.

Procellariiformes Fürbringer, 1888.

Procellariidae Leach, 1820.

*Puffinus* Brisson, 1760.

*Puffinus* cf. *griseus* Gmelin, 1789.

#### Referred materials

**Figure 4:**
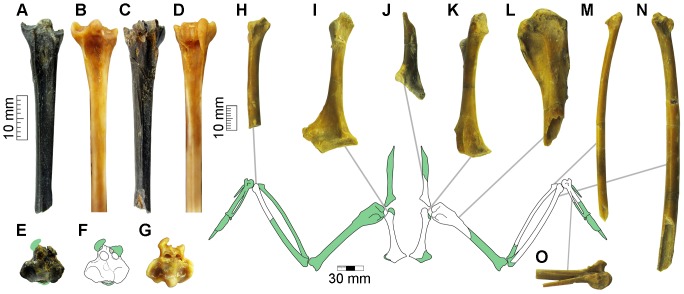
Fossil shearwater and cormorant from the Horcon Formation. Proximal fragment of a fossil left tarsometatarsus assigned as *Puffinus* cf. *griseus* (SGO-PV 21490) in (**A**) dorsal, (**C**) plantar and (**E**) proximal views; and (**F**) sketch showing the details of the proximal surface. Proximal section of the same element in the extant great shearwater (*Puffinus gravis*) in (**B**) dorsal, (**D**) plantar and (**G**) proximal views. Partial wings and pectoral girdle of the small cormorant *Phalacrocorax* sp. (SGO-PV 21443): (**H**) fragment of right ulna in dorsal view; (**I**) right coracoid in dorsal view; (**J**) proximal fragment of left scapula in medial; (**K**) left coracoid in ventral view; (**L**) proximal fragment of left humerus in caudal view; (**M**) left radius lacking of proximal end in ventral view; (**N**) left ulna lacking of proximal end in ventral view; and (**O**) proximal fragment of left carpometacarpus in ventral view.

SGO-PV 21490, proximal fragment of left tarsometatarsus (Figure 4A,C,E,F). Collected from layer 12.

#### Measurements

Maximum length preserved, 43.8 mm; proximal mediolateral width, 9.7 mm; dorsoplantar width of shaft at the middle point, 4.29 mm.

### Anatomical comparison

In dorsal view, the medial cotyla is medially expanded, giving an asymmetric appearance to the proximal end of the tarsometatarsus as in *P. griseus*, *P. gravis* and *P. bulleri*, but weaker than in *P. pacificus*. In genera such as *Calonectris*, *Pterodroma* and *Pterodromoides* the proximal end is more symmetrical. The medial cotyla is also located more proximally than the lateral cotyla as is usual in *Puffinus*, whereas in *Calonectris* both cotylae are of subequal height. The medial margin of the medial cotyla is strongly pointed as in *Puffinus* and *Calonectris*. The intercotylar prominence is slightly damaged and is relatively wide as in *P. griseus* and *P. bulleri*, being smaller that in *P. gravis* and *P. creatopus* and less rounded that in *P. pacificus*. As in *P. griseus*, *P. gravis* and *P. pacificus* the dorsal infracotylar fossa is completely open, whereas in *P. creatopus*, *Calonectris* and *Bulweria* the retinacular extensors scar (impressio retinacula extensorii) forms a bridge that covers the medial foramen. In *P. bulleri* the retinacular extensors scar forms a smaller ridge proximal to the medial foramen.

The hypotarsal crests are partially preserved. In proximal view, well-defined lateral and medial canals can be identified. The presence of both canals is typical of *Puffinus*, whereas a lateral canal partially close and a medial sulcus can be seen in genera like *Pterodroma* and two shallow sulci are present in *Fulmarus*.

The middle shaft is strongly mediolaterally compressed as in *Puffinus*, whereas in genera like *Calonectris*, *Bulweria*, *Pterodroma*, *Pterodromoides* and *Pachyptyla* the shaft is more expanded mediolaterally. From the middle point of the shaft to the proximal end, the shaft gradually expands mediolaterally as in *P. griseus*, *P. creatopus* and *P. bulleri*; whereas in *P. gravis*, *P. pacificus* and *Calonectris* the width of the shaft is more constant, expanding proximally only at the level of the proximal foramina. The dorsal sulcus is shallow and poorly defined as in *Puffinus*, whereas in genera like *Pterodroma* and *Pterodromoides* it is deep and well delimited by medial and lateral ridges.

### Remarks

This specimen is equivalent in size and morphology to the extant *Puffinus griseus*, one of the largest shearwaters and a much larger taxon than most of the fossils previously attributed to *Puffinus* in the Southeastern Pacific [44]. It represents a species smaller than *P. gravis* and *Procellaria*, similar in size to *Calonectris* and larger than *Puffinus creatopus*, *P. bulleri* and *P. pacificus*. Nevertheless, considering the fragmentary nature of this record and the intraspecific and interspecific variation within the extant species [44], we avoid a more specific assignment.

All the specimens described by Stucchi and Urbina [44], from the Miocene of the Pisco Formation represent species similar in size or smaller than *P. bulleri* and *P. pacificus*. Additionally, an isolated neurocranium from the Late Miocene of the Bahia Inglesa Formation has been attributed to *Puffinus* [45], and represents a shearwater larger than *P. griseus* and *P. creatopus*. This specimen possibly belongs to the same taxon as the skull found in the Pisco Formation and erroneously attributed to *Fulmarus* by Cheneval [5]. Other Pliocene records of *Puffinus* in the Eastern Pacific include the extinct Early Pliocene species *P. tedfordi* [46] from Baja California, Mexico, and *P. fethami* [47] from California, USA; and the Late Pliocene *P. kanakoffi* [47] and *P. gilmorei* [48] from San Diego, USA. All these species are smaller than *P. griseus*.

Suliformes (Sharpe, 1891).

Phalacrocoracidae Reichenbach, 1850.

*Phalacrocorax* Brisson, 1760.

*Phalacrocorax* sp.

#### Referred materials

SGO-PV 21443, associated wings and pectoral girdle elements including left and right coracoids, proximal fragment of right scapula, proximal fragment of right humerus, right ulna lacking of proximal end, right radius lacking of proximal end, distal fragment of left ulna, proximal fragment of right carpometacarpus (Figure 4H–O); SGO-PV 21446, proximal fragment of left carpometacarpus. SGO-PV 21443 collected from layer 12; SGO-PV 21446 collected from layer 9.

#### Measurements

SGO-PV 21443, left coracoid, maximum length preserved 53.6 mm; left coracoid, sternal facet width 16.4 mm; right coracoid, maximum length preserved 57.1 mm; right scapula, proximal width 14.2 mm; right humerus, maximum proximal width 20.1 mm; right ulna, maximum length preserved 103.8 mm; right carpometacarpus, proximal anteroposterior width 12 mm.

### Remarks

These specimens represent a cormorant smaller than *Phalacrocorax bougainvilli* and similar in size to *P. gaimardi* and *P. brasilianus*. This range is equivalent to that described for *Phalacrocorax* sp. from the Late Miocene and Pliocene of the Pisco Formation [5], [7], and the Late Pliocene of the La Portada Formation [16]. Similar specimens are also known from the Late Miocene of the Bahia Inglesa Formation, including an associated braincase and sternum [18], [49]. A second and larger species, *P.* aff. *bougainvillii*, is also known from the Late Miocene and Pliocene of the Pisco and Bahia Inglesa formations [2], [7], [18].

As has been mentioned by previous authors [7] and based on the differences in the proportions respective to extant Pacific cormorants, these specimens probably represent an extinct species. Despite the fact that SGO-PV 21443 is one of the most complete specimens of seabird currently known from Chile, here we avoid naming a new species, considering the availability of more complete specimens in the Pisco Formation. This unnamed taxon was the most common cormorant along the coast of Chile and Peru during the late Neogene.

## Discussion

### The South American Record of seabirds during the Pliocene

**Figure 5:**
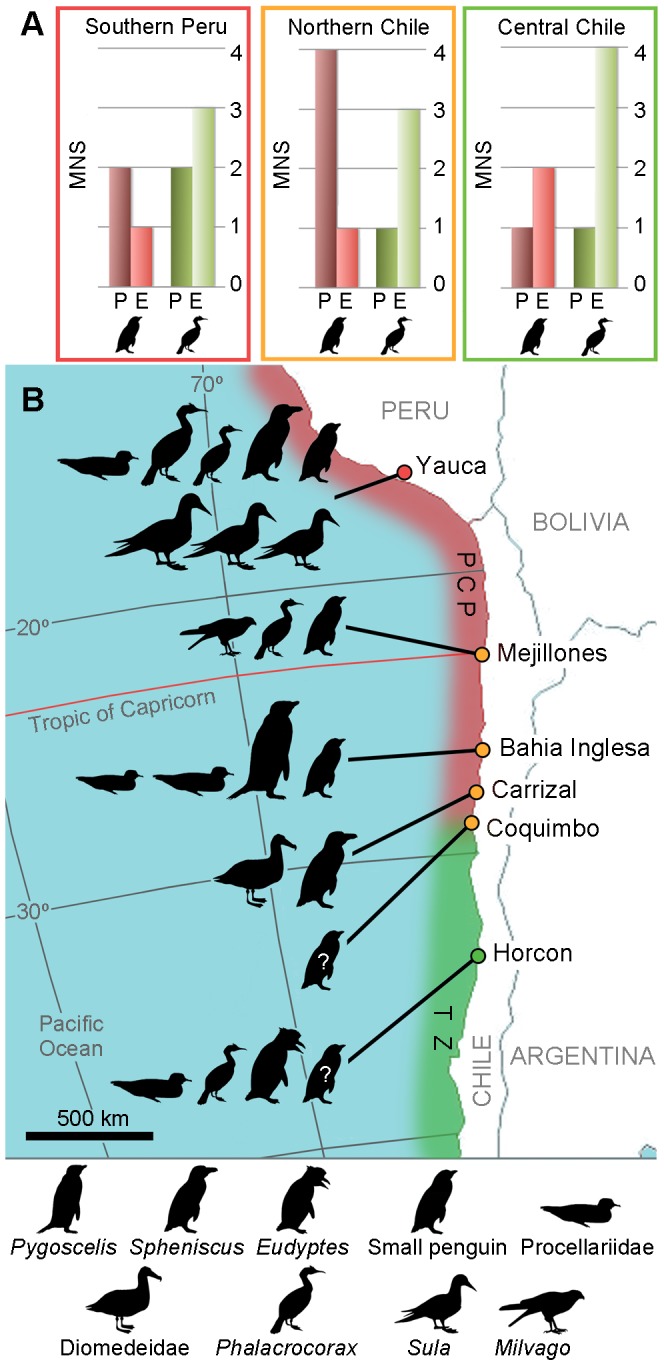
The Pliocene record of birds in the Southeast Pacific. **A.** Comparison between the Pliocene (P) and extant (E) richness of penguins and cormorants for each major area represented as minimum number of species (MNS); **B.** Map showing the main fossiliferous localities, the zoogeographical regions (PCP, Peruvian–Chilean Province; TZ, Transition Zone) and the species recorded.

The fossil record of seabirds during the Pliocene in the Southeastern Pacific has been mostly restricted to the area currently comprised by the PCP, and the Horcon assemblage is the first fauna described for the transitional zone and the southernmost seabird locality currently known for the Pliocene of South America (Figure 5B).

After the recent publication of new radiometric dates for the vertebrate horizons Sacaco and Sacaco Sur from the Pisco Formation [50], the only possibly Pliocene locality from Southern Peru is Yauca [51], [52]. If this age can be confirmed, this locality will be the most diverse seabird assemblage for this interval in South America, including penguins, cormorants and petrels, along with at least three species of boobies currently unrecorded in other areas [7], [51], [53]. A minimum of three other localities is known in Northern Chile. These are the outcrops of the La Portada Formation in Mejillones [16], the Pliocene Lechero Member of the Bahia Inglesa Formation at Los Negros locality [37], [54], and the Carrizal locality of the Coquimbo Formation [18]. It is possible that the record from Coquimbo [17] also belongs to the Pliocene levels of the Coquimbo Formation. Penguins are abundant and diverse across these localities, whereas the record of other families is comparatively rare and includes petrels, albatrosses, cormorants and caracaras.

Although the Pliocene record consists exclusively of extant families, the seabird assemblages from Chile and Peru are composed of a mixture of modern and extinct taxa. In comparison with the Late Miocene, the only suprageneric taxon currently absent from the Pliocene record is the Pelagornithidae, whereas the great booby *Sula magna* persists in Southern Peru [51], and the small cormorant *Phalacrocorax* sp. is still present from Peru to the transitional zone [7], [16]. It is possible that only one of the up to five Late Miocene species of penguins persisted until the Pliocene: the medium-sized *Spheniscus* sp. recorded in Yauca and Carrizal [18], [53]. On the other hand, at least three extant species are possibly present during the Pliocene: the Humboldt penguin *Spheniscus humboldti* [53], the Guanay cormorant *Phalacrocorax bougainvillii* [7], and the sooty shearwater *Puffinus* cf. *griseus* recorded in Horcon. The first two species are endemic to the PCP, whereas the last one has a wide dispersion range but only breeds in Subantarctic regions including the Magellanic region in South America [10]. This suggests that the processes that originated the modern seabird faunas along the Pacific coast of South America had already started in the Late Pliocene; however, most of them remain minor elements in the Pliocene assemblages.

Comparing the current and Pliocene richness of penguins and cormorants across the PCP and the transition zone (Figure 5A), we can see a general increase in the case of the cormorants, and a decrease in the diversity of penguins in Peru and Northern Chile. Similarly to the situation described for the North Pacific prior the Pliocene [55], cormorants are comparatively rare in the record of Peru and particularly Chile. Two species are known from the Late Miocene to the Pliocene, whereas no fewer than three are currently present across the Humboldt System. The small Neogene cormorant *Phalacrocorax* sp., a possible ecological analogue of the Red-legged Cormorant *P. gaimardi*, is by far the most common cormorant until the Late Pliocene, whereas specimens identifiable as the Guanay cormorant *P. bougainvillii* are scarce. In contrast, the Guanay cormorant is currently one of the most abundant and main guano-producing species in the PCP [56], and it is also the most abundant species in Pleistocene sites of southern Peru [57]. This change in dominance is most likely related to the warmer oceanic conditions during the Pliocene [58], and their effect on the main prey of the Guanay: the Peruvian anchovy. Warm periods, like El Niño events, have a negative effect on the population of anchovy and drive the alternate regimen shifts between this species and the sardine [59]. Drops in the anchovy population have strong effects on the population of the Guanay cormorant and, in combination with commercial fishing, had been associated with the recent decline of that species [60]. The predominance of warmer waters in the Pliocene of Northern and Central Chile (see [61]) represents an adverse scenario for these species.

On the other hand, only two species of penguins remained in Peru by the Pliocene and a minimum of four were present across Northern Chile, whereas the Humboldt penguin is the only species that currently inhabits this area. This decrease in the diversity of penguins is not well recorded in Horcon, where two species of banded penguins can currently be found and only one species of crested penguin can be confirmed for the Pliocene. Small penguins in the size range of the Humboldt penguin became more common in Northern Chile, and remains of medium sized *Spheniscus* are comparatively rare during the Pliocene, whereas slightly larger species like *S. urbinai* were common across the PCP during the Late Miocene. However, at least one species, “*Pygoscelis*” *grandis*, reached a large size in the range of *Aptenodytes*. This relatively high diversity of penguins is intriguing considering the warmer conditions described for the Pliocene [58], [61], [62]. Nevertheless, there is also a reduction in size and a drop in the richness of species with respect to the Late Miocene, where a minimum of four species can be found in the Bahia Inglesa bonebed [3], [18]. Interestingly, no more than two species have been found at the same locality in the Pliocene in Northern Chile.

### Subantarctic seabirds during the Pliocene Warming

The possible presence of genera currently restricted to the Subantarctic and Antarctic in lower latitudes is another enigmatic characteristic of seabird faunas during the Pliocene. Despite the existence of other Neogene species originally attributed to the Antarctic genera *Pygoscelis* [37], [63], [64] and *Aptenodytes* [63], *Eudyptes calauina* is the only one currently supported by phylogenetic analysis as part of a Subantarctic genus (see [41]). The presence of subadults and possibly juveniles indicates that this was a breeding species in Central Chile. Currently, the Southern Rockhopper and Macaroni penguins are the only two breeding species in South America, with colonies restricted to austral islands over 50°S [65]. Nevertheless, most of the crested penguins breed on islands surrounding New Zealand between 35 and 55°S [66], in waters with average Sea Surface Temperatures (SST) between 15 and 7°C. Multiproxy reconstructions and climate modeling suggest SST around 16°C during the austral winter and 20°C during summer in central Chile during the Pliocene [67]. However, there is currently no Pliocene data available that comes directly from the Humboldt Current System. The living crested penguins are migratory and highly seasonal breeders [66]. This suggests that *E. calauina* was a migratory species, probably breeding during the austral winter like the Northern Rockhopper and the Fiordland penguin [68]; and adapted to warmer conditions than the species that currently inhabit South America, being probably more similar to the New Zealand species. This may also be one of the causes for the disappearance of this species from the transitional zone with the beginning of Quaternary cooling.

The Benguela Current system in South Africa is the only other area with a well-known Pliocene record of seabirds in the South Hemisphere [1], [30], [69]. Seals [70] and a high diversity of penguins [30] and Procellariiformes, including prions (*Pachyptila*) and diving petrels (*Pelecanoides*) [71], are known for this region and have been interpreted as evidence of Subantarctic conditions [1], [69]. This seems to contradict the warmer temperatures currently known for the Pliocene [58], [61], [62], [67]. However, it is possible that the meaning of this vertebrate assemblage in terms of cooler temperatures has been overstated, being also congruent with temperate conditions. Seals are a dominant element of the pinniped assemblages during the Neogene in the Southern Hemisphere [54], being diverse under the warmer climate of the Miocene. Despite the fact that prions mainly breed in Subantarctic islands, many species, like the Fairy and Broad-billed prions [72], also breed in temperate areas. Furthermore, the Magellanic Diving-petrel is the only diving petrel truly restricted to cold-temperate regions, whereas two of the remaining species reach warm-tempered areas and one, the Peruvian Diving-petrel, is completely restricted to the PCP [72]. Finally, multiproxy reconstructions suggest SST around 18°C during winter and 22°C during summer in the Western Cape of South Africa for the Late Pliocene [67], being a similar range to the current summer conditions at the area.

There is no doubt that, as in South America, the fossil assemblages of South Africa show significant differences with respect to the faunas that currently inhabit the region. Furthermore, the South African seabird assemblage during the Pliocene was apparently more similar to the extant assemblage of the South American PCP. Boobies (*Sula*), the dominant sulid genus during the Neogene and the only one still present in South America, were also present during the Pliocene in South Africa and later replaced by the Cape gannet *Morus capensis* [1], [69]. Diving petrels and several species of storm petrels have been identified as endemic species present in the Humboldt system without an ecological equivalent in the present-day Benguela system [73]; whereas at least one species of diving petrel and two of storm petrels were present during the Pliocene [71]. The disappearance of these cold/temperate elements from South Africa is intriguing considering the cooling trend of this region. Current estimations suggest that the drop in SST was stronger and faster in the Benguela than in the Humboldt system: approximately 8°C in 3.3 Ma versus 4°C in 3.8 Ma [62]. One of the main drivers proposed for this faunal turnover is the change in the availability of breeding areas (islands and/or beaches) due to sea level fluctuation [1], [30], [54], [71]. This is certainly a possible explanation for a global reduction in the richness of Procellariiformes and penguins, which largely prefer islands to continental beaches. On the other hand, the differential extinction of cold/temperate birds in the Benguela and Humboldt systems can be related to the larger latitudinal thermal gradient in South America, which allows these taxa to expand or contract their distribution more easily than in Southern Africa. In this sense, the mixed seabird fauna of Horcon shows that at least some seabirds species, like the small Neogene cormorant, where spread across a wide range of climatic conditions during the Pliocene. Additionally, it is possible that the difference in the SST cooling rate [62] could also play a role in this differential extinction.

### Conclusion

The fossil record of Horcon reflects the existence of a mixed seabird fauna in central Chile during the Pliocene, resembling the current assemblages from the transitional zone. This area is unique through the presence of *E. calauina*, the oldest record of this genus, which is currently absent from the Humboldt System but present in the Magellanic region. It also includes the first Pliocene record of the sooty shearwater that currently breeds in the Magellanic region, and a small cormorant shared with Southern Peru and Northern Chile. The presence of a transitional zone in central Chile during the Pliocene is congruent with the comparatively cooler conditions suggested for Southern Chile based on foraminifera [74], and the annual temperature oscillation that could affect the area according to multiproxy reconstructions [67]. This thermal gradient could also play an important role in the preservation of a higher diversity of cold/temperate seabirds in the Humboldt Current, compared with similar upwelling systems like the Benguela Current (west coast of southern Africa). Nevertheless, it is clear that despite the latitudinal differences across the Humboldt System, the Pliocene seabirds represent a distinctive assemblage linking the living faunas with the Late Miocene forms. At the moment, the lack of Neogene records in Southern Chile prevents us from making more detailed comparisons with the Magellanic region, but it is expected that this gap can be filled in the near future.

## Supporting Information

Document S1 **Complementary information, Figures S1–S3 and Table S1.** Stratigraphic column. Expanded anatomical description. Phylogenetic analysis including details about the changes introduced to the original data set, GenBank accession numbers and authorships, list of morphological characters, and list of osteological synapomorphies. Figure S1: Location map and stratigraphic column of the Horcon Formation. Figure S2: Detail of bone surface texture in penguin specimens. Figure S3: Complementary strict and Adams consensus trees from combined and morphology-only analyses. Table S1: Comparative measurements for the tarsometatarsus.
(PDF)

Dataset S1 **Phylogenetic analysis matrix.**
(NEX)
