# Supplementary material for: The Evolution of Seabirds in the Humboldt Current: New Clues from the Pliocene of Central Chile
Source: PLoS One. 2014 Mar 12;9(3):e90043. doi: 10.1371/journal.pone.0090043 (PMC3951197; doi:10.1371/journal.pone.0090043)
Supplement: Document S1 — Complementary information, Figures S1–S3 and Table S1. Stratigraphic column. Expanded anatomical description. Phylogenetic analysis including details about the changes introduced to the original data set, GenBank accession numbers and authorships, list of morphological characters, and list of osteological synapomorphies. Figure S1: Location map and stratigraphic column of the Horcon Formation. Figure S2: Detail of bone surface texture in penguin specimens. Figure S3: Complementary strict and Adams consensus trees from combined and morphology-only analyses. Table S1: Comparative measurements for the tarsometatarsus. (PDF) [file pone.0090043.s001.pdf]

Document S1 - Chávez Hoffmeister et al. The evolution of seabirds in the Humboldt Current: New clues from the Pliocene of central Chile

Stratigraphic column

Two well-defined stratigraphic intervals can be identified across the entire sequence. The lower unit crops out with an estimated thickness of less than 8 m and consists of a sequence of conglomerates and greenish glauconitic sandstones containing some white calcareous concretions. The presence of carbonized plant remains and the absence of macrofossils are also characteristic of this unit. The upper unit corresponds to the main section of the sequence. The lithology of this unit is characterized by layers of fine to coarse sandstone, light-colored and poorly consolidated, which are interspersed with few conglomeritic layers.

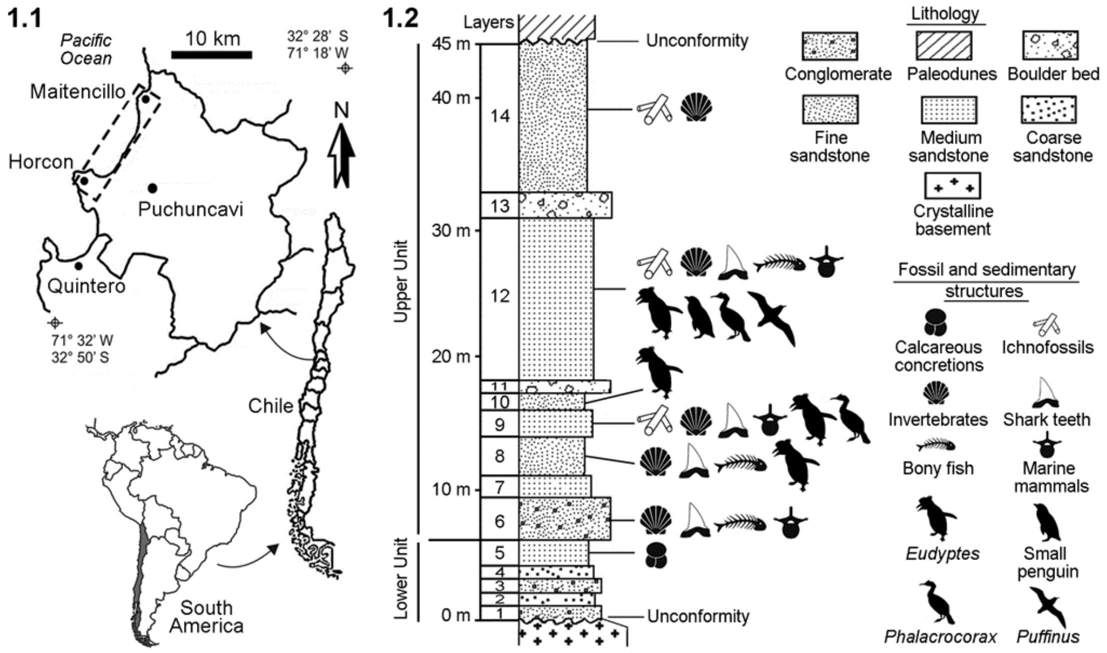

Figure S1.1. Location map. **S1.2.** Stratigraphic column of the Horcon Formation.

Expanded anatomical description

Vertebrae

SGO-PV 21452 is most likely a fourth cervical. Its general shape resembles those observed in *Eudyptes chrysocome*, *E. chrysolophus* and *Madrynornis*. The dorsal tori are strongly prominent, relatively narrow anteroposteriorly and with their extremities pointing laterocaudally as in the compared species of *Eudyptes* and *Madrynornis*; whereas in *Spheniscus urbinai* the tori of module 3 and 4 (sensu Guinard et al. 2010) are less prominent, more rounded and wider anteroposteriorly. The hypapophysis is larger than in compared species of the genus, relatively slender and ventrocaudally directed.

Humerus

Based on SGO-PV 21449 and the section preserved in SGO-PV 21451, the head is strongly prominent proximally and has the shape of a rampant arch with the apex ventrally located in posterior view; whereas in *Eudyptula* and *Spheniscus* the head is less prominent, giving a flatter appearance to the proximal extreme of the humerus. In SGO-PV 21451, a notch between the dorsal tubercle and the base of the humeral head is clearly visible in caudal view (Figure 1d). This notch is usually absent in *Spheniscus*, *Eudyptula* and *Tereingaornis.* In SGO-PV 21449, the capital incisure is completely separated from the transverse ligament sulcus as in *Eudyptes*, *Spheniscus* and *Eudyptula* (Figure 1e). However, these are connected by a sulcus in *Aptenodytes*, *Inguza*, *Madrynornis*, *Palaeospheniscus* and *Eretiscus.* A deep pit for ligament insertion is present on the proximal surface adjacent to the head as in most Spheniscidae. This pit is

absent or extremely shallow in *Pygoscelis*, *Madrynornis*, *Palaeospheniscus*, *Eretiscus* and occasionally in *Aptenodytes patagonicus.* The proximal margin of the tricipital fossa is weakly projected in proximal view as in most living and Neogene penguins, like *Madrynornis*, *Inguza* and *Palaeospheniscus*. In contrast, it develops a lip-like projection that is well exposed in proximal view in extant species of *Spheniscus* and occasionally in *Pygoscelis antarctica*. Unlike the living species of *Eudyptes* and *Spheniscus chilensis*, where the proximal border of the tricipital fossa forms a symmetrical concavity in ventral view; in *E. calauina* it is asymmetrical and slightly concave. The impressio insertii m. supracoracoideus and m. latissimus dorsi are separated by a small gap as in Spheniscidae. The shaft robustness index (SRI: proximodistal length / ventrodorsal width at middle point, see Character 176) shows a value (4) at the limit between most of the living penguins (4.1-4.9) and the bulkiest fossil taxa such as *Paraptenodytes robustus* (MACN A-11032, holotype of *Isotremornis nordenskjoldi*) (3.9), *Pachydyptes* (3.8) and *Platydyptes novaezealandiae* (3.6). The nutrient foramen is situated on the ventral face of the shaft as in *Palaeospheniscus* and all Spheniscidae, whereas in *Madrynornis* and *Eretiscus* it is situated on the anterior face. The dorsal edge of the shaft is curved and without a clear preaxial angle as is usual in *Eudyptes*, nevertheless, a well-defined angle can occasionally be found as the normal condition in *Aptenodytes*, *Pygoscelis*, *Megadyptes*, *Spheniscus*, *Palaeospheniscus* and *Eretiscus*. The posterior trochlear ridge reaches the ventral edge of the shaft. This condition is also present in living species of *Eudyptes*, *Megadyptes* and *Palaeospheniscus*; where as a result, the ridge often slightly exceeds the ventral margin in cranial view but not in caudal view. In *Aptenodytes, Pygoscelis* and *Madrynornis* the ridge extends beyond the ventral margin, but does not

reach the ventral edge in *Inguza, Eretiscus* and most species of *Spheniscus*. The trochlear angle measured in SGO-PV 21451, defined as the angle between the main axis of the shaft and the tangent of ventral and dorsal condyles, is equal to 45°. This angle is within the range of most Spheniscidae (greater than or equal to 45°), although it is smaller in *Inguza* (43°), *Madrynornis* (41°) and *Tereingaornis* (41°); and occasionally in *Spheniscus urbinai* (43°-52°), *Palaeospheniscus* (39°-49°) and *Eretiscus* (44°-54°). In ventral view, the ventral condyle is almost parallel to the dorsal condyle and does not extend the anterior edge of the humerus which is flattened as in most Spheniscidae. In *Madrynornis*, *Palaeospheniscus* and *Eretiscus* the ventral condyle is more rounded and slightly surpasses the anterior edge. The dorsal end of the scapulotricipital sulcus is curved caudally and completely separated from the humerotricipital sulcus by the medial trochlear ridge; as in *Aptenodytes*, *Spheniscus muizoni*, *Madrynornis*, *Palaeospheniscus* and *Eretiscus*. In other species of *Eudyptes* and *Spheniscus,* the dorsal end of the scapulotricipital sulcus is dorsally connected to the humerotricipital sulcus.

Tibiotarsus

The proximal fragment of a tibiotarsus SGO-PV 21447 (Figure 1h) partially resembles that of *S. megaramphus*. However, it is much smaller than in that species and many of their similarities are shared with other Spheniscidae, including *Eudyptes*. Similar to *Spheniscus* or *Aptenodytes*, the lateral crest is strongly prominent cranially in lateral view, unlike *Eudyptes* where the cranial edge of the crest is almost aligned with the cranial edge of the diaphysis. The cranial margin of the lateral crest is thickened but delimited by the proximal and distal edges of the crest shaft, whereas in *Spheniscus,*

Eudyptes, *Madrynornis* and *Palaeospheniscus* the margin is often projected proximally creating a proximal prominence in lateral view. The lateral longitudinal flexor fossa is relatively well developed and clearly delimited medially by a longitudinal crest, whereas the medial fossa of the collateral ligament is shallow and medially open as in most of the extant penguins. In *S. muizoni* and *Spheniscus* sp. MUSM 800 the collateral ligament fossa is deep and medially enclosed by a short proximal ridge. In the distal fragment of tibiotarsus SGO-PV 21488 (Figure 1i) the sulcus extensorius is located close to the midline of the shaft. The distal epiphysis is relatively wide mediolaterally to a similar degree as in living species of *Eudyptes* and less than in *Megadyptes*. The tubercle for the retinaculum of the fibularis muscle is well defined and prominent in caudal view. As in *Eudyptes*, *S. chilensis*, *Madrynornis* and *Palaeospheniscus*; a tuberosity for the extensor reticulum appears in the lateral edge forming a shallow lip-like crest. This tuberosity is usually absent in *Spheniscus* and occasionally present in *Aptenodytes* and *Pygoscelis* (Göhlich, 2007). The caudal edge of the medial condyle is slightly damaged. However, the edge is apparently continuous also in medial view, unlike in *S. chilensis* and *Eudyptes* where it is distally notched (Göhlich, 2007). In cranial view, the lateral condyle is slightly inflated laterally as in *Eudyptes* and *Madrynornis* creating a relatively straight lateral edge.

Tarsometatarsus

The surface texture on the holotype SGO-PV 21487 suggest that it can be attributed to an adult, whereas the porous and fibrous textures observed on the tarsometatarsus SGO-PV 21444 allow us to attribute these specimen to a subadult (Figure S2.A-B).

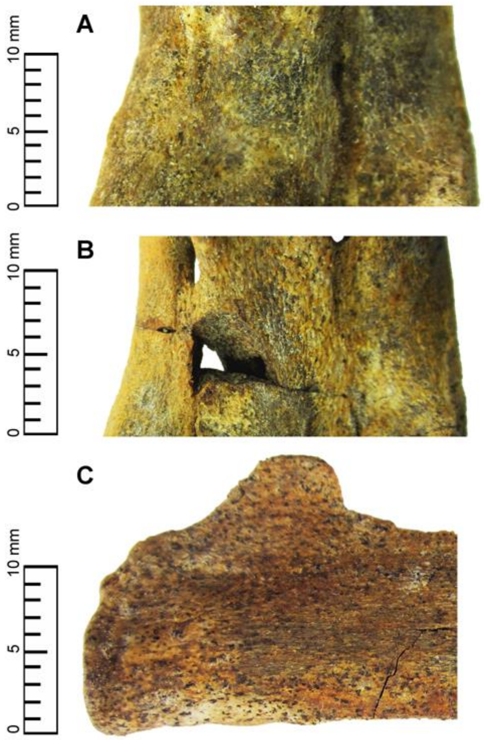

Figure S2. Detail of bone surface texture in penguin specimens. The bones in adults have a smoother surface as can be observed in the holotype of *Eudyptes calauina* SGO-PV 21487 (**A**, plantar). In contrast, the porous and fibrous textures observed on the tarsometatarsus SGO-PV 21444 (**B**, plantar) and the ulna SGO-PV 21455 (**C**, ventral), along with the degree of development of some anatomical features; allow us to attribute these specimens to subadults.

The collateral lateral ligament scar is extremely shallow in SGO-PV 21444 (Figure 1p) and absent in SGO-PV 21487 (Figure 1j) as in *Eudyptes*, *Megadyptes* and *Aptenodytes*. This scar is located proximodorsally in *Spheniscus*, *Eudyptula*, *Inguza*, *Nucleornis*, *Madrynornis*, *Palaeospheniscus* and *Eretiscus;* creating a truncate proximolateral vertex in dorsal view. The intermediate hypotarsal crest is indistinguishable from the lateral crest in plantar view as in all Spheniscidae, while in

Madrynornis, *Palaeospheniscus*, *Eretiscus* and occasionally in *Eudyptula* an extremely shallow groove is visible in proximal view slightly delimited by both crests. As in *Eudyptes*, *Madrynornis*, *Palaeospheniscus*, *Eretiscus* and *Korora*, the lateral hypotarsal crest is well defined in SGO-PV 21487 (Figure 1k) and forms a diagonal ridge that overhangs the lateral foramen, that is slightly less defined in SGO-PV 21444 (Figure 1q). The medial proximal vascular foramen opens plantarly at the medial surface of the medial hypotarsal crest as in *Eudyptes*, *Spheniscus*, *Eudyptula* and *Madrynornis*; whereas in *Pygoscelis* and *Nucleornis* it opens directly at the plantar surface. In SGO-PV 21487 the foramen is relatively large as in *E. chrysolophus* and *Madrynornis*; and is even larger in SGO-PV 21444, being visible in plantar view as in *Megadyptes* and occasionally in *E. chrysocome*. This degree of development is rare in *Spheniscus*, being often smaller as in *Eudyptula*. The lateral proximal vascular foramen is small as in most Spheniscidae, with the exception of *Spheniscus* in which it is occasionally enlarged (this is particularly common in *S. humboldti*). In contrast, the lateral foramen is vestigial in *Eretiscus* and completely absent in *Nucleornis*. The medial dorsal sulcus is moderately deep as in *Eudyptes*, *Megadyptes*, *S. urbinai* and *S. chilensis*. In *E. schlegeli* and *E. sclateri* the sulcus is occasionally shallow as in *Aptenodytes*, *Pygoscelis*, *Madrynornis*, *Palaeospheniscus* and *Eretiscus*. In contrast, in *Eudyptula*, most of the *Spheniscus* species, *Inguza* and *Nucleornis* the sulcus is much deeper. The lateral edge of the metatarsus IV in SGO-PV 21487 is strongly compressed dorsoplantarly creating a sharp edge in lateral view (Figure 1m), as in *Palaeospheniscus*. In plantar view, the lateral intertrochlear notch is proximally deeper than the medial as in *Eudyptes*, the extant *Spheniscus* spp., *S. muizoni*, *Eudyptula*, *Madrynornis* and *Palaeospheniscus*. The

trochlea metatarsi IV is shorter than the trochlea metatarsi II in dorsal view as in *Eudyptes*, *Madrynornis* and *Palaeospheniscus*; whereas in *Aptenodytes*, *Pygoscelis*, *Megadyptes*, *Eudyptula* and *Spheniscus* both are sub-equal. In distal view, the dorsal edges of the trochlea metatarsi III and IV are aligned at the same plane (Figure 1o,u), whereas in extant species of *Spheniscus*, *S. chilensis* and *S. megaramphus*, the trochlea IV is displaced dorsally. Plantarly, the lateral ridge of the trochlea IV is much larger than the medial ridge in distal view, creating a plantar edge that is strongly pointed laterally and flattened medially as in *Eudyptes*, *Pygoscelis*, *S. chilensis*, *S. urbinai*, *S. megaramphus*, *Eudyptula*, *Madrynornis* and *Palaeospheniscus*. Also in distal view, the plantar edge of the trochlea II is slightly deflected plantarly with respect to the plane defined by the most plantar point of the trochleae III and IV (Figure 1o,u), as in *Eudyptes*, *Aptenodytes*, *Pygoscelis*, the extant species of *Spheniscus*, *S. chilensis*, *S. urbinai*, *S. muizoni* and *Madrynornis.* In plantar view, the trochlea II is slightly pointed with parallel medial and lateral edges in SGO-PV 21487 as in *Megadyptes*, *Aptenodytes*, *Eudyptula*, *S. megaramphus*, *Madrynornis* and *Paleospheniscus.* However, in extant species of *Eudyptes* the trochlea II is strongly pointed with a more rounded medial edge or following the medial edge of the tarsometatarsus. An intermediate state can be seen in SGO-PV 21444, where the trochlea II is slightly pointed but the medial edge follows the medial edge of the tarsometatarsus.

Taxon	Specimens	Length	Proximal width	Distal width	Elongation index (EI)
Eudyptes calauina	SGO-PV 21487	41.3	24.3	27.5	1.7
Eudyptes calauina	SGO-PV 21444	41	22.7	25.3	1.8
Eudyptes chrysolophus	(n = 1) [a]	25.1	14.2	17	1.7
Eudyptes moseleyi	Z.2007.065	30.9	15.3	20.5	2
Eudyptes chrysocome	(n = 3) [a]	29	14.8	19.5	1.9
Eudyptes robustus	(n = 18) [b]	29.36	14.73	19.35	1.9
Eudyptes pachyrhynchus	(n = 28) [b]	30.02	15.2	19.75	1.9
Eudyptes sclateri	(n = 19) [b]	31.6	17.03	22	1.8
Eudyptes schlegeli	AMNH 5399	33.3	18.7	22.2	1.8
Megadyptes antipodes	(n = 20) [b]	35.03	18.37	22.94	1.9
Spheniscus humboldti	(n = 2) [a]	35.7	16.6	21.2	1.8
Spheniscus magellanicus	(n = 5) [c]	31.4	15.7	19.72	2
Spheniscus demersus	(n = 8) [a]	32.7	15.6	19.6	2
Spheniscus muizoni	MNHN PPI 147	33.31	16.22	21.53	2
Spheniscus urbinai	MUSM 401	48.76	24.69	30.09	1.9
Spheniscus megaramphus	MUSM 2087	50.97	26.62	34.66	1.9
Eudyptula minor	(n = 3) [a]	22	10.2	13.4	2.1
Pygoscelis adeliae	(n = 18) [c]	32.48	17.09	21.03	1.9
Pygoscelis papua	(n = 9) [c]	32.84	19.31	24.1	1.7
Pygoscelis antarctica	(n = 3) [c]	28.93	16.07	20.3	1.8
Pygoscelis grandis	SGO-PV 1104	49.2	27.33	–	1.8
Aptenodytes patagonicus	(n = 3) [1]	45.5	27.8	33.3	1.6
Aptenodytes forsteri	(n = 3) [1]	45.2	32.3	38.1	1.1
Madrynornis mirandus	MPEF PV 100	36.3	18.1	19.1	2
Inguza predemersus	SAM PQ L23018	27.2	12.8	–	2.1
Nucleornis insolitus	SAM PQ MBD3	40.4	22.4	–	1.8
Palaeospheniscus bergi	(n = 5) [c]	36.4	16.54	20.56	2.2
Palaeospheniscus patagonicus	(n = 3) [c]	40.06	19.07	23.33	2.1
Palaeospheniscus biloculata	(n = 5) [c]	42.1	19.1	24.1	2.2
Eretiscus tonnii	MLP 81-VI-26-1	19.6	8.16	–	2.4

Table S1. Comparative measurements for the tarsometatarsus in millimeters. Sources of measurements: **a.** Stephan (1979), **b.** Worthy (1997), **c.** Acosta Hospitaleche and Gasparini (2007).

Phylogenetic analysis

Modifications to the matrix

During the revision of the original matrix of Ksepka et al. (2012), some inconsistencies between the list of characters and the coded state in the matrix were detected. The state code of characters 91 and 103 as defined in the character list is switched with respect to the states in the matrix; so that the state 0 in the matrix is defined as state 1 in the list and vice versa. Character 98 defines a state 2 that is not used in the matrix. The same happens with the state 0 of character 187. On the other hand, characters 136, 173 and 195 include an extra state in the matrix that is not defined in the character list. Finally, six characters (201, 202, 203, 204, 207 and 208) have different numerations between the list of characters and the matrix. None of these errors had an impact on the original results offered by Ksepka et al. (2012), but they might create serious mistakes when new taxa are added. All these errors have been corrected for the present version and have been also corrected in an update of Ksepka et al. (2012) (see Dryad database).

GenBank molecular sequences

GenBank accession numbers

Taxon	12S	16S	COI	Cyt-*b*	RAG-1
A. forsteri	DQ137187	DQ137147	DQ137185	DQ137225	DQ137246
A. patagonicus	AY139221	DQ137148	DQ137186	AY 138623	DQ 137247
D. capense	X82517	—	—	AF076046	—
D. exulans	DQ137205	DQ137165	DQ137168	DQ137208	DQ137229
E. chrysocome	AY139630	—	DQ525796	—	DQ525776
E. chrysolophus	DQ137197	DQ137157	DQ137171	AF076052	DQ137223
E. filholi	DQ525741	—	DQ525781	—	DQ525761
E. moseleyi	DQ525746	—	DQ525786	—	DQ525766
E. pachyrhynchus	U88007, X82522	DQ 137152	DQ137170	DQ137210	DQ137231
E. robustus	DQ137193	DQ137153	DQ137176	DQ137126	DQ137237
E. schlegeli	DQ137196	DQ137156	DQ137175	DQ137215	DQ137236
E. sclateri	DQ137194	DQ137154	DQ137169	DQ137309	DQ137230
E. minor	NC_004538	DQ137164	DQ137174	NC_004538	DQ137235
G. immer	AF173577	DQ137166	DQ137167	DQ137207	DQ137288
G. stellata	AF173587	AY293618	AY666477	AF158250	—
M. giganteus	X82523	—	—	AF076060	—
M. antipodes	DQ137198	DQ137158	DQ137184	DQ137224	DQ1372245
O. oceanicus	—	—	DQ433048	AF076062	—
O. leucorhoa	—	—	AY666284	AF0706064	—
P. desolata	—	—	—	AF076068	—
P. urinatrix	X82518	—	—	AF076076	DQ881818
P. immutabilis	—	—	DQ433933	PIU48949	—
P. palpebrata	—	—	—	U48943	DQ881822
P. aequinoctialis	—	—	—	U74350	—
P. brevirostris	NC007174	NC007174	NC007174	NC007174	—
P. gravis	AF175572	AF173752	DQ434014	U74354	—
P. adeliae	AF173573	DQ137149	DQ137183	DQ137223	DQ137224
P. antarctica	DQ137190	DQ137150	DQ137181	AF076089	DQ137242
P. papua	DQ137191	DQ137151	DQ137182	AF076090	DQ137243
S. demersus	DQ137199	DQ137159	DQ137177	DQ137217	DQ137238
S. humboldti	DQ137201	DQ137161	DQ137180	DQ137220	DQ137241
S. magellanicus	DQ137200	DQ137160	DQ137178	DQ137218	DQ137239
S. mendiculus	DQ137202	DQ137162	DQ137179	DQ137219	DQ137240
T. melanophrys	AY158677	AY158677	NC_007172	U48955	AY158677

Authorship

12S rDNA: Baker *et al.* (2006): DQ137187, DQ137190–1, DQ137193–4, DQ137196–202, DQ137205; Banks *et al.* (2006): DQ525741, DQ525746, DQ525756; Cooper & Penny (1997): U88007, U88024; García-Moreno *et al.* (unpublished): AY139621, AY139623, AY139630; Stanley & Harrison (1999): X82517–8, X82522–3, X82533; Slack *et al.* (2006): AY158677; NC_004538; Paterson *et al.* (1995): AF173573, AF173577–8. **16S rDNA:** Baker *et al.* (2006): DQ137147–62, DQ13714765–6; Van Tuinen *et al.* (2000): AY158677, AY293618. **Cytochrome *b*:** Stanley & Harrison (1999): DQ137207–10, DQ137215–20, DQ13723–5, AF158250; Baker *et al.* (2006): DQ525761, DQ525766, DQ525776, NC_004538; Nunn *et al.* (1996): U48943, U48949, U48955; Nunn & Stanley (1998): AF076051–2, AF076046, AF076060, AF076062, AF076064, AF076068, AF076076, AF076089–90, U74335, U74350, U74353. **COI:** Nunn & Stanley (1998): DQ137167–72, DQ137174–86; Baker *et al.* (2006): DQ525781, DQ525786, DQ525796; Hebert *et al.* (2004): AY666477, AY666284; Kerr *et al.* (2007): DQ433048; Slack *et al.* (2006): NC_007172. **RAG-1:** Baker *et al.* (2006): DQ137230–3, DQ137235–47; Ericson *et al.* (2006): DQ881818, DQ881822.

Morphological character descriptions

List based on KF. Citations for the primary source of the characters are indicated with abbreviations as follows. A = Ando (2007); AH = Acosta Hospitaleche *et al.* (2007); BG = Bertelli & Giannini (2005); C = Clarke *et al.* (2007); CL = Clarke *et al.* (2010); GB = Giannini & Bertelli (2004); K = Ksepka *et al.* (2006); KC = Ksepka & Clarke (2010); KF = Ksepka *et al.* (2012); KT = Ksepka & Thomas (2011); OH = O'Hara (1986). Citation of Figures is also offered for some characters. Characters that are new or have been modified significantly from previous studies are indicated.

Integument

1. Tip of mandibular rhamphotheca, profile in lateral view: pointed (0); slightly truncated (1); strongly truncated, squared off (2); truncated but with a rounded margin (e.g., as seen in Procellariiformes) (3). (GB1)

2. Longitudinal grooves on the base of the culmen: absent (0); present (1). (GB2)

3. Longitudinal grooves on the base of latericorn and ramicorn: absent (0); present (1). (GB3)

4. Feathering of maxilla, extent: totally unfeathered (0); slightly feathered, less than half the length of maxilla (1); feathering that reaches half the length of maxilla (2); feathering surpassing half the length of maxilla (3). (GB4) **Ordered**

5. Ramicorn, inner groove at tip: absent (0); present and single (1); present and double (2). (GB5) **Ordered**

6. Orange or pink plate on ramicorn: absent (0); present (1). (GB6)

7. Plates of rhamphotheca, inflated aspect: absent (0); present (1). (GB7)

8. Gape: not fleshy (0); margin narrowly fleshy (1); margin markedly fleshy (2). (GB8) **Ordered**

9. Ramicorn color pattern: black (0); red (1); pink (2); yellow (3); orange (4); green (5); blue (6). (GB9)

10. Latericorn and ramicorn, light distal mark: absent (0); present (1). (GB10)

11. Latericorn color: black (0); red (1); orange (2); yellow (3); green (4); blue (5). (GB11)

12. Culminicorn color: black (0); red (1); orange (2). (GB12)

13. Maxillary and mandibulary unguis, color: black (0); red (1); yellow (2); green (3); blue-gray (4). (GB13)

14. Ramicorn, ultraviolet color spot (reflectance peak): absent (0); present (1). (KC14)

15. Bill of downy chick, color: dark (0); reddish (1); pale, variably horn to yellow (2); blue (3). (GB14)

16. Bill of immature, color: dark (0); bicolored red and black (1); red (2); yellow (3); gray (4). (GB15)

17. External nares: present (0); absent (1). (GB17)

18. Nostril tubes in adult: absent (0); present (1). (GB16)

19. Nostril tubes in hatchling: absent (0); present (1). (GB16)

20. External nares: well-separated (0); fused at midline (1). (KC19)

21. Iris color: dark (0); reddish-brown (1); claret red (2); yellow (3); white (4); silvery gray (5). (GB18)

22. Scale-like feathers: absent (0); present (1). (GB19)

23. Rhachis of contour feathers: cylindrical (0); flat and broad (1). (GB20)

24. Rectrices: form a functional fan (0); do not form a fan (1). (GB21)

25. Remiges: differentiated from contour feathers (0); indistinct from contour feathers (1). (GB22)

26. Apteria: present (0); absent (1). (GB23)

27. Molt of contour feathers: gradual (0); simultaneous (1). (GB24)

28. Yellow pigmentation in crown feathers (pileum): absent (0); present (1). (GB25)

29. Head plumes (crista pennae): absent (0); present (1). (GB26)

30. Head plumes (crista pennae), aspect: compact (0); sparse (1). (GB27)

31. Head plumes (crista pennae), aspect: directed dorsally (0); directed posteriorly, not drooping (1); directed posteriorly, drooping (2). (GB28)

32. Head plumes (crista pennae), position of origin: at base of bill close to gape (0); on the recess between latericorn and culminicorn (1); on forehead (2). (GB29) **Ordered**

33. Head plumes (crista pennae), color: yellow (0); orange (1). (GB30)

34. Nape (occiput), crest development: absent (0); slight (1); distinct (2). (GB31) **Ordered**

35. Periocular area, color: black (0); white (1); yellow (2); bluish gray (3). (GB32)

36. Fleshy eyering: absent (0); present (1). (GB33)

37. White eyering: absent (0); present (1). (GB34)

38. White eyebrow (supercilium): absent (0); narrow, from postocular area (1); narrow, from preocular area (2); wide, from preocular area (3). (GB35) **Ordered**

39. Loreal area (lorum), aspect: feathered (0); with spot of bare skin in the recess between latericorn and culminicorn (1); with spot of bare skin contacting eye (2); bare skin extending to the base of bill (3). (GB36) **Ordered**

40. Auricular patch (regio auricularis): absent (0); present (1). (GB37)

41. Throat pattern: black (0); white (1); yellow (2); irregularly streaked (3); with chinstrap (4). (GB38)

42. Collar: absent (0); at most slight notch present (1); present, diffusely demarcated (2); black, strongly demarked (3). (GB39) **Ordered**

43. Breast, golden in color: absent (0); present (1). (GB40)

44. Dorsum color: black (0); dark bluish gray (1); light bluish gray (2). (GB41)

45. Black marginal edge of dorsum between lateral collar and axillary patch, contrasting with dorsum: absent (0); present (1). (GB42)

46. Black dots irregularly distributed over white belly: absent (0); present (1). (GB43)

47. Flanks, dark lateral band reaching the breast: absent (0); present (1). (GB44)

48. Distinct dark axillary patch of triangular shape: absent (0); present (1). (GB45)

49. Flanks, extent of dorsal dark cover into the leg: incomplete, not reaching tarsus (0); complete, reaching tarsus (1). (GB46)

50. Rump: indistinct in color from dorsum (0); distinct white patch (1). (GB47)

51. Tail length: short, the quills barely emerge from the rump (0); quills distinctly developed (1). (GB48)

52. Outer rectrices, color: same as inner rectrices (0); lighter than inner rectrices (1). (GB49)

53. White line connecting leading edge of flipper with white belly: absent (0); present (1). (GB50)

54. Flipper, upperside, light notch at base: absent (0); present (1). (GB51)

55. Leading edge of flipper, pattern of upperside: black (0); white (1). (GB52)

56. Leading edge of flipper, pattern of underside: white (0); incompletely dark (1); completely dark and wide (2). (GB53)

57. Flipper, underside, dark elbow patch: absent (0); present (1). (GB54)

58. Flipper, underside, tip pattern: immaculate (0); patchy, in variable extent (1); small circular dot present (2). (GB55)

59. Immature plumage, white eyebrow (supercilium): absent (0); or present (1). (GB56)

60. Immature plumage, throat pattern (jugulum): black (0); or mottled (1); or white (2); or brown (3). (GB57)

61. Immature plumage, flanks, dark lateral band: absent (0); or present (1). (GB58)

62. Chicks hatch almost naked: no (0); yes (1). (GB59)

63. Dominant color pattern of first down: pale gray (0); distinctly brown (1); bicolored, dark above and whitish bellow (2); uniformly blackish gray (3). (GB60)

64. Dominant color pattern of second down: pale grey (0); distinctly brown (1); bicolored, dark above and whitish bellow (2); uniformly blackish gray (3). (GB61)

65. Chick, second down, collar: absent (0); present (1). (GB62)

66. Feet, dorsal color: dark (0); pink (1); orange (2); white-flesh (3); blue (4). (GB63)

67. Feet, soles distinctly darker than dorsal surface: absent (0); present (1). (GB64)

68. Feet, unguis digiti: flat (0); compressed (1). (BG65)

Reproductive Biology

69. Clutch size: two eggs (0); one egg (1). (GB65)

70. Incubatory sac: absent (0); present (1). (GB66)

71. Nest: no nest, incubation over the feet (0); nest placed underground, either burrowed in sand or inside natural hollow or crack (1); open nest, a shallow depression on bare ground or in midst of vegetation (2). (GB67)

72. Size of first egg relative to the second egg: similar (0); dissimilar, first smaller (1); dissimilar, second smaller (2). (GB68)

73. Crèche: absent (0); small, 3-6 birds (1); formed by dozens to hundreds of immatures (2). (GB69)

74. Eggs, shape: oval (0); conical (1); spherical (2). (BG71)

75. Ecstatic display: absent (0); present (1). (BG72)

Osteology

76. Premaxilla, tip (rostrum maxillare): pointed (0); weakly hooked (1); strongly hooked (2). **Ordered**. **NOTE**: In state 2, the tip ventrally exceeds the level of the tomial edge; whereas in state 1 the tip is approximately at the same level as the tomial edge. (GB0) (OH: fig.4)

77. Premaxilla, frontal process, naso-premaxillary suture: visible (0); obliterated (1). (BG95) (BG: fig.12)

78. Nasal cavity, external naris (cavum nasi, apertura nasi ossea), caudal margin respect the rostral margin of the hiatus orbitonasalis (fossa antorbitalia): overlapping each other (0); non overlapping (1). (OH5) (OH: fig.2; BG: fig.11)

79. Internarial bar (pila supranasalis), dorsal view: slender, slightly constricted laterally (0); wide throughout its length (1). (OH6) (OH: fig.3; BG: fig.12)

80. Internarial bar (pila supranasalis), shape in cross section: suboval (0); inverted U-shape (1). (C75)

81. Internarial bar (pila supranasalis), profile in lateral view (culmen): dorsal edge curves smoothly to tip of beak (0); pronounced step in dorsal edge (1). (KC78)

82. Tomial edge (crista tomialis), plane of tomial edge respect to the basitemporal plate (lamina parasphenoidalis): approximately at the same level (0); dorsal to the level of the basitemporal plate (1). (BG97)

83. Lacrimal: unperforated (0); perforated (1). (OH11) (OH: Fig.2; BG: Fig.11)

84. Lacrimal: reduced, concealed in dorsal view (0); small portion exposed in dorsal view (1); well-exposed in dorsal view (2). (BG82) **Ordered**

85. Lacrimal, contact with frontal: suture (0); fusion (1). (KT89)

86. Lacrimal, dorsal process: closely applied to the nasal (0); rostral arm of dorsal process separated from the nasal by a slit-like rostro-caudally elongate opening (1). This character originally referenced the frontal, however the actual separation occurs along the nasal-lacrimal contact (modified in KT90). (BG83)

87. Frontal, shelf of bone bounding salt-gland fossa (fossa glandulae nasalis) laterally: absent (0); present (1). (OH10) (BG: fig.9)

88. Squamosal, temporal fossa (fossa temporalis), size: fossae separated by considerable wide surface (at least the width of the cerebellar prominence) (0); more extensive, fossae meeting or nearly meeting at midline of the skull (1). (BG76) (BG: fig.9; K: fig.5)

89. Squamosal, temporal fossa (fossa temporalis), depth of caudal region: flat (0); shallow (1); greatly deepened (2). (BG77) (BG: fig.10) **Ordered**

90. Squamosal, development of the opening that transmits the a. ophthalmica externa in the caudoventral area of the temporal fossa (near nuchal crest): small or vestigial (0); well-developed (1). (BG78) (BG: fig.10)

91. Supraoccipital, paired grooves for the exit of v. occipitalis externae (sulcus vena occipitalis externae): poorly developed (0); deeply excavated (1). (BG74) (BG: fig.8)

92. Orbit, fonticuli orbitocraneales: small or vestigial (0); broad and conspicuous openings (1). (BG79) (BG: fig.10)

93. Ectethmoid: absent (0); weakly developed, widely separate from the lacrimal (1); well developed, contacting or fused to the lacrimal (2). (BG80)

94. Basioccipital, subcondylar fossa (fossa subcondylaris): absent or shallow (0); deep (1). (BG73) (BG: fig.7)

95. Basitemporal plate (lamina parasphenoidalis), dorsoventral position with respect to the occipital condyle: ventral to the level of the condyle (0); at the level of the condyle (1); dorsal to the level of the condyle, surface depressed (2). (BG86) **Ordered**

96. Basipterygoid process (proccessus basipterygoideus): absent (0); vestigial or poorly developed (1); well developed (2). (BG87) (BG: fig.7) **Ordered**

97. Eustachian tubes (tuba auditiva): open or very little bony covering near the caudal end of the tube (0); mostly enclosed by bone (1). (BG88) (BG: fig.7)

98. Pterygoid, shape: elongated (0); slight lateral expansion of rostral end (1) rostal end broad, pterygoid sub-triangular (2). (BG89) (BG: fig.7; KC: fig.22) **Ordered**

99. Palatine, lamella choanalis: curved and smooth plate, slightly differentiated from main palatine blade (0); ridged, distinct from main blade by a low keel (1); extended vertically ventrally forming the crista ventralis (2). (BG90) (BG: fig.13) **Ordered**

100. Vomer, laterally compressed, vertical laminae and free from palatines (0); horizontally flattened laminae and ankylosed with palatines (1). **NOTE**: This character defines a state 2 in KT98 and KF98. However, this state does not appear in any of the included taxa. (BG91) (BG: fig.13)

101. Facial foramen (foramen n. facialis): absent (0); present (1). (BG92)

102. Jugal arch, bar shape in lateral view: straight (0); slightly curved (1); ventrally bowed (2); strongly curved, sigmoid shape (3). (BG93) (BG: fig.14) **Ordered**

103. Jugal arch, dorsal process: absent (0); present (1). This pointed process is located on the caudal end of the jugal, adjacent to the condyle for articulation with the quadrate. (BG94)

104. Quadrate, relative lengths of otic and orbital processes (processus oticus and processus orbitalis): otic process longest (0); orbital process longest (1). (KC102)

105. Quadrate, otic process (processus oticus), rostral border, tubercle for m. adductor mandibulae externus, pars profunda: absent (0); present, as a ridge (1); presence, as a tubercle (2). (BG96) (BG: fig.15)

106. Quadrate, otic process (processus oticus), rostral border, tubercle for m. adductor mandibulae externus, pars profunda: contiguous with squamosal capitulum (0); separated from squamosal capitulum (1). (KC104) (KC: fig.23)

107. Quadrate, processus oticus, caudal margin in lateral view: straight (0); flexed so as to be concave caudally (1). (A9)

108. Mandible, symphysis: extensive bony connection (0); short terminal bony connection (1). (C101)

109. Mandible, posteriorly projected midline spur from dentary underlying symphysis: absent (0); present (1). (KC107)

110. Mandible, coronoid process (processus coronoideus), position on the dorsal margin of the mandible with respect to caudal mandibular fenestra (fenestra mandibulae caudalis): markedly rostral (0); on the rostral end of the fenestra (1); caudal to fenestra (2). (BG98) (BG: fig.16) **Ordered**

111. Mandible, rostral fenestra (fenestra mandibulae rostralis): imperforate or small opening (0); large opening (1). (OH8) (OH: fig.4; BG: fig.16)

112. Mandible, caudal fenestra (fenestra mandibulae caudalis): open, can be seen through from the medial or lateral aspects (0); nearly or completely concealed by the splenial medially (i.e., fenestra not visible in the medial aspect) (1). (OH9)

113. Mandible, mandibular ramus: depth subequal over entire ramus (0); pronounced deepening at midpoint (1). (BG101) (BG: fig.16)

114. Mandible, mandibular ramus: essentially straight or gently sloping (0); pronounced ventral deflection near midpoint (1). (KC112)

115. Mandible, dentary, length of dorsal edge relative to mandibular ramus length in lateral view: markedly more than half the length of ramus (0); approximately half the length of ramus (1). (BG103)

116. Mandible, articular, medial process (processus medialis): not hooked (0); hooked (1). (BG104) (BG: fig.17; K: fig.6)

117. Mandible, angular, aspect in dorsal view: sharply truncated caudally (0); caudally projected, forming retroarticular process (processus retroarticularis) (1). (BG106) (BG: fig.17)

118. Mandible, angular, retroarticular process (processus retroarticularis), aspect in dorsal view in relation to the articular area for the quadrate between the lateral and medial condyles (condylus lateralis and condylus medialis): broad, approximately equal to the articular area (0); moderately long, narrower than the articular area (1); very long, longer and narrower than the articular area (2). (BG105) (BG: fig.17) **Ordered**

119. Mandible, medial emargination between medial and retroarticular processes (processus retroarticularis and processus medialis): absent (0); weak concavity (1); strong concavity (2). (K108) (K: fig.6) **Ordered**

120. Atlas, processus ventralis: absent or slightly developed (0); well developed, high and prominent ridge on the dorsal surface of the arcus atlantis (1). (BG108) (BG: fig.18)

121. Transition to free cervicothoracic ribs begins at: 13th cervical vertebrae: (0); 14th cervical vertebrae (1); 15th cervical vertebrae (2). (BG109) **Ordered**

122. Cervical vertebrae, transverse process (processus transversus) in last five cervical vertebrae: not elongated laterally (0); greatly elongated laterally (1). (BG111)

123. Thoracic vertebrae, posteriormost vertebrae: heterocoelous (0); weakly opisthocoelous; (1); strongly opisthocoelous (2). (K114) **Ordered**

124. Thoracic vertebrae, deep excavation on lateral face of posterior thoracic vertebrae: absent (0); present (1). (KC124)

125. Synsacrum, number of incorporated vertebrae: nine (0); eleven (1); twelve (2); thirteen (3); fourteen (4), fifteen or more (5). (C117)

126. Synsacrum, height of crista synsacri between acetabuli: flat or weakly projected (0); strongly projected (1). (KC126)

127. Synsacrum, first incorporated vertebra, position of fovea costalis: caudal to level of processus transversus (0); cranial to level of transverse process (1). (KF229)

128. Synsacrum, ventral surface of first few incorporated vertebrae: rounded or flattened (0); sharp, blade-like ventral margin (1). (A63)

129. Caudal vertebrae: seven (0), eight (1), nine (2). (BG113) **Ordered**

130. Pygostyle, shape: tapers to a narrow edge both dorsally and ventrally as in most volant birds (0); triangular in cross-section with a wide, flat ventral margin (1). (KF232)

131. Thoracic ribs, uncinate processes (costae, processes uncinati): elongate, narrow (0), wide at base, spatulate (1), extremely wide at base (2). Reference to bifurcation of the processes in state 2 from previous formulations of this character has been removed, as bifurcation shows individual variation in all species of *Pygoscelis.* (BG114) (BG: fig.19)

132. Thoracic ribs, uncinate processes (costae, processes uncinati): fused to ribs (0); unfused (1) (KC129)

133. Sternum, external spine (spina externa rostri): absent (0); present (1). (OH13) (BG: fig.20)

134. Sternum, facies articularis furculae projects as a distinctive process: absent (0); present (1). (BG116) (BG: fig.20)

135. Sternum, articular facets for coracoids (sulcus articularis coracoideus): meet or overlap one another at midline (0); separated by wide non-articulatory surface (1). (C122) (BG: fig.20)

136. Sternum, orientation of sulcus articularis coracoideus in ventral view: sulci oriented in essentially straight horizontal line (0); sulci directed caudolaterally so as to together form an inverted U shape (1). (A15) (KF: fig.3)

137. Sternum, labrum internum: continues as sharp ridge onto the base of the spina externa (0); fades away without continuing onto the base (1). (C123)

138. Sternum, caudal incisurae: absent (0); two (1); four (2). (KC134)

139. Sternum, trabecula lateralis projects caudal to main body of sternum: no (0); yes (1). (KF234)

140. Furcula, hypocleidium (apophysis furculae): absent or low knob-like process (0); long, blade-like process (1). (BG117)

141. Furcula, ramus: sub-ovoid in cross-sectional omal end (0); mediolaterally flattened and craniocaudally expanded at omal end (1). (CL218)

142. Scapula, acromion: craniodorsally directed, nearly parallel to long axis of scapular shaft at apex (0); forms a blunt triangular projection with apex directed approximately at 45 degree angle from long axis of scapular shaft (1); narrow and tapering, apex omally directed (2); narrow and tapering, apex directed at a right angle to scapular shaft (3). **NOTE**: In KF136, only three states are defined. However, the matrix includes four states. Here the four states of that matrix are used. (CL223)

143. Scapula, facies articularis humeralis: rounded, projecting from shaft of scapula (0); compressed and ovoid, projecting from shaft of scapula (1); flattened and nearly merged with shaft of scapula (2). (KF235)

144. Scapula, blade, caudal half (corpus scapulae, extremitas caudalis): blade-like (0); slightly expanded (1); broadly expanded, paddle-shaped (2). (BG118) (KC: fig.24)

145. Coracoid, length: shorter than humerus (0); greatly elongated, longer than humerus (1). (KC137)

146. Coracoid, processus acrocoracoideus, region of tuberulum brachiale: craniocaudally compressed (0); craniocaudally expanded, with a large flat surface cranial to tuberulum brachiale. (A22)

147. Coracoid, scapular cotyle (scapula cotylaris): deep and socket-like (0); shallow depression (1). (CL217)

148. Coracoid, medial margin, coracoidal fenestra: complete (0); incomplete (1); absent (2). (OH14) (OH: fig.6; BG: fig.21; K: fig.7)

149. Coracoid, foramen nervi supracoracoidei: absent (0), present (1). Mayr (2005) cited ontogenetic evidence that this foramen is not homologous to the coracoidal fenestra of penguins. (K122)

150. Coracoid, sternal margin (extremitas sternalis coracoidei): greatly expanded (0); moderate expansion (1). (BG120) (BG: fig.21)

151. Coracoid, profile of the sternal margin (extremitas sternalis coracoidei) in ventral view: convex (0) concave (1), flat (2). **NOTE**: State 1 describes the strongly concave margin seen in *Gavia* and the slight concavity seen in *Aptenodytes*, whereas most penguins show a flatter margin and are consequently are coded as 2. This differs from the

code used by K124, KC141, KT143 and KF143, who use state 1 for all extant penguins. (K124) (K: fig.7; KC: fig.25)

152. Coracoid, lateral process (processus lateralis): absent or highly reduced (0); well-developed (1). (KC142)

153. Coracoid, facies articularis sternalis, dorsal surface: single facet (0); two facets (1). (KF236)

154. Forelimb elements: subcircular in cross section (0); flattened (1). (BG121)

155. Humerus, head: very developed and reniform, continuous with tuberculum dorsale: absent (0); present (1). (BG122)

156. Humerus, proximal edge of head in posterior view: semicircular humeral head with apex located near midline (0); humeral head with the shape of a rampant arch, with ventral apex, slightly prominent proximally (1); humeral head with the shape of a rampant arch with ventral apex, strongly prominent proximally (2). **NEW STATE**: State 1 refers to the degree of prominence seen in *Spheniscus*, whereas state 2 can be seen in *Pygoscelis*. Both states are included in state 1 of C132, KC145, KT147 and KF147. (C132)

157. Humerus, notch between the dorsal tubercle and humeral head: present (0); absent (1). **NEW CHARACTER**: This character is clearly visible in caudal view. State 0 can be seen in *Pygoscelis* and state 1 in *Spheniscus.* See Göhlich (2007).

158. Humerus, incisura capitis: essentially confluent with sulcus transversus (0); connected with the sulcus transversus trough a narrow sulcus (1); completely separated from sulcus transversus (2). **NEW STATE**: State 1 refers to the condition seen in *Palaeospheniscus patagonicus*, whereas state 2 can be seen in *Eudyptes*. This connection

is more subtle than in *Anthropornis* in which the transverse sulcus and capital incisure forms a single cranial sulcus (state 0). (K127) (K: fig.10)

159. Humerus, capital incisure: extends to secondary tricipital fossa (0); separated from secondary tricipital fossa (1). (CL222)

160. Humerus, pit for ligament insertion on proximal surface adjacent to head: absent or very shallow (0); deep (1). (K128) (K: fig.8)

161. Humerus, orientation of intumenscentia humeri and tuberculum ventrale: intumenscentia projects ventrally from shaft, tuberculum oriented posteriorly (0); intumenscentia projects ventrally from shaft, tuberculum oriented ventrally (1); intumenscentia projected more anteroventrally (so as to be partially obscured in posterior view), tuberculum oriented anteroventrally (2). (K129) (K: fig.10)

162. Humerus, proximal margin of tricipital fossa (fossa tricipitalis): weak projection (0); projects so as to be well-exposed in proximal view (1). (K135) (K: fig.8)

163. Humerus, proximal border of tricipital fossa in ventral view: concave proximal margin (0), straight to slightly concave border (1). **NOTE**: State 0 represents a truly concave margin as can be seen in *Madrynornis*; whereas state 1 includes the slightly concave margin usually present in *Palaeospheniscus* and the almost straight one present in *Spheniscus*. (KT154) (KT: fig.1m-o)

164. Humerus, tricipital fossa (fossa tricipitalis), aspect: small with penetrating pneumatic foramina (0); moderate fossa without pneumatic foramen (1); deep fossa without pneumatic foramen (2). (BG123)

165. Humerus, tricipital fossa (fossa tricipitalis): single (0); bipartite (1). (BG 124) (BG: fig.22)

166. Humerus, deltoid crest, impressio m. pectoralis: superficial poorly-defined groove (0); shallow, well-defined oblong fossa (1); deep, well-defined oblong fossa (2). **Ordered**. **NOTE**: In state 2, the deltoid crest is well defined distally, whereas in state 1 it is poorly defined. (BG125)

167. Humerus, impressio insertii m. supracoracoideus: small, semicircular scar (0); greatly elongated with long axis sub-parallel to main axis of humeral shaft (1). (K133) (K: fig.9)

168. Humerus, impressio insertii m. supracoracoideus and m. latissimus dorsi: separated by a wide gap (0); separated by a moderate gap (1); separated by small gap or confluent (2). (K134) (K: fig.9; KC: fig.26) **Ordered**

169. Humerus, coracobrachialis caudalis scar: clearly separated from head (0); scar contacts distal margin of head (1). (CL219)

170. Humerus, coracobrachialis caudal scar: deeply depressed, subcircular (0); flat, ovoid, oriented dorsoventrally (1); flat, elongate and oriented obliquely at approximately 45 degree angle to long axis of shaft (2). (CL220)

171. Humerus, groove for coracobrachialis nerve: absent or poorly defined (0); sharp, narrow sulcus (1). (CL221)

172. Humerus, shaft, dorsoventral width: shaft thins or maintains width distally (0); shaft widens distally (1). (K136) (K: fig.10)

173. Humerus, nutrient foramen (foramen nutricum): situated on ventral face of shaft (0) situated on anterior face of shaft (1). (C143)

174. Humerus, anterior face of shaft elongate depression near ventral margin: absent (0); present (1). (C144)

175. Humerus, shaft, sigmoid curvature: absent or weak (0); strong (1). (K137) (K: fig.10)

176. Humerus, shaft robustness index (proximodistal length / ventrodorsal width at middle point): greatly elongated, SRI ≥ 7 (0); greatly slender, 7 > SRI ≥ 6 (1); slender, 6 > SRI ≥ 5 (2); thick, 5 > SRI ≥ 4 (3); bulky, DRI < 4 (4). **NEW CHARACTER**: For this index, the proximodistal length is measured from the contact between the dorsal tubercle and humeral head (proximal end), to the contact between the ulnar condyle and the trochlear processes (distal end). The ventrodorsal width is measured at the middle point of the diaphysis, regardless of the position of the preaxial angle. State 0 can be seen in *Waimanu*, state 1 in *Perudyptes*, state 2 in *Palaeeudyptes*, state 3 in *Palaeospheniscus* and state 4 in *Pachydyptes*. (KC: fig.26)

177. Humerus, preaxial angle: absent or inconspicuous (0); well defined (1). **NEW CHARACTER**: Although there is a large quantitative variation in the development of this angle, its pattern of presence or absence is a stable character among many taxa. In state 0 the dorsal edge of the shaft is curved and without a clear preaxial angle, whereas in state 1 the angle creates a clear inflection point. State 0 can be seen in *Eudyptes* and state 1 in *Spheniscus.*

178. Humerus, development of dorsal supracondylar tubercle (processus supracondylar dorsalis): absent (0); compact tubercle (1); elongate process (2). (BG126)

179. Humerus, demarcation of sulcus scapulotricipitalis: not demarcated (0); passage a well-marked groove (1); development of trochlear ridge for articulation with os sesamoideum m. scapulotricipitis (2). (BG127). **Ordered**

180. Humerus, posterior trochlear ridge: extends beyond ventral margin of the humeral shaft (0); reaches the ventral margin (1); does not reach the ventral edge (2). **NEW STATE**: The new state 1 can be seen in *Palaeospheniscus* and *Eudyptes*. As a result, the ridge often slightly exceeds the ventral margin in cranial view but not in caudal view. (BG128) (BG: fig.23)

181. Humerus, scar for origin of m. brachialis: ovoid fossa on cranial face of humerus at distal end (0); proximodistally elongate scar on dorsal margin of humeral shaft, with diagonally oriented proximal border (1); proximodistally elongate scar on dorsal margin of humeral shaft, with chevron-shaped proximal border (2). (A34)

182. Humerus, angle between main axis of shaft and tangent of ulnar and radial condyles (condylus dorsalis and condylus ventralis): less than 35° (0); 35° to 45° (1); greater than or equal to 45° (2); nearly 90° (3). **NEW STATE**: The new state 0 can be seen in *Perudyptes* and *Anthropornis*. State 3 refers to the state in most flying birds, and state 2 represents values closer to 45 than to 90°. States 0 and 1 were included in state 0 of KC141, KT169 and KF169. The values of angles for fossil taxa were obtained by photo analysis using TpsDIG version 2. (K141) (KC: fig.26)

183. Humerus, ulnar condyle (condylus ventralis): rounded condyle displaced over the anterior edge of the humerus (0); ulnar condyle almost parallel to the radial, slightly surpassing the anterior edge of the humerus (1); ulnar condyle almost parallel to the radial, not surpassing the anterior edge of the humerus (2). **MODIFIED**: Under this new definition, state 0 can be seen in *Palaeeudyptes,* state 1 in *Palaeospheniscus* and state 2 in *Spheniscus*. Although the anterior projection of the ulnar condyle in state 1 is less

pronounced than in state 0 (seen in the most basal penguins), it is more pronounced than in state 2. (K142) (K: fig.11)

184. Humerus, shelf adjacent to condylus ventralis: large, ratio of condyle width: shelf width >1.3 (0); moderate, ratio of condyle width: shelf width 1.3-2.0 (1); greatly reduced, less than half condyle width (2). (K143) (K: fig.11) **Ordered**

185. Radius, shaft: narrow (0); broad and flattened (1). (KC166)

186. Radius, proximally projecting spike-like process at cranial margin: absent (0); present (1). (KF239)

187. Ulna, olecranon position: arises at level of or proximally surpassing humeral cotylae (0); slightly distally displaced from cotylae (1); located one fourth of length to proximal end (2). **MODIFIED**: Because a combination of shapes and positions can be seen in penguins, we decided to separate both in two independent characters, previously coded together (K144). Under this new definition, state 0 can be seen in *Puffinus*, state 1 in *Icadyptes* and state 2 in *Spheniscus*. (K144) (K: fig.12; KC: fig.27)

188. Ulna, olecranon shape: short and robust (0); tab-like projection with a rounded posterior margin (1); tab-like projection with a squared posterior margin (2); tab-like projection with a distinctive angular posterior margin (3). **NEW CHARACTER**: Separation of the position and shape of the olecranon into two independent characters, previously coded together (K144). State 0 can be seen in *Puffinus*, state 1 in *Icadyptes*, state 2 in *Kairuku* and state 3 in *Spheniscus*. (K: fig.12; KC: fig.27)

189. Ulna, distinct process extending toward sulcus humerotricipitalis of humerus: absent (0), present (1). (K145)

190. Ulna incisura radialis: concave in proximal view, so that the ulna contacts the proximal radius at both its caudal and ventral surfaces (0); obsolete, so radius and ulna abut one another at a nearly flat contact (1). (KF240) (KF: fig.5g-h)

191. Ulnare: U-shaped (0); triangular, fan-shaped wedge (1). (KC169)

192. Ulnare, distal angle: rounded (0); pointed (1). **NOTE**: This character refers to the distal angle in the specialized fan-shaped ulnare of penguins and is considered non-comparable for outgroup taxa. (KF241)

193. Carpometacarpus, pisiform process (processus pisiformis): well-projected round tubercle (0); reduced to a low ridge (1). (C155)

194. Carpometacarpus, distal facet on metacarpal I: absent (0); present (1). (C156) (KC: fig.28)

195. Carpometacarpus, metacarpal II, distinct anterior bowing: absent (0); present (1). (C157)

196. Carpometacarpus, extension of metacarpals II and III: subequal or III slightly shorter (0); metacarpal III projects markedly distal of metacarpal II. (C158) (KC: fig.28)

197. Carpometacarpus, metacarpal III, distal articular surface (facies articularis digitalis major): wedge shaped or broadens anteriorly in distal view (0), slightly depressed ovoid surface (1). (C159)

198. Carpometacarpus, extensor process (processus extensorius): present (0); absent (1) (KC175)

199. Carpometacarpus, metacarpal II, distal expansion: absent (0); present (1). (KC176)

200. Phalanges of manus, phalanx digit III proximal process: absent (0); present (1). (BG130) (KC: fig.28)

201. Phalanges of manus, relative length of phalanx III-1 and phalanx II-1: phalanx III-1 shorter (0); subequal (1). (C161) (KC: fig.28)

202. Phalanges of manus, length relative to carpometacarpus: long (0); short (1). (BG131)

203. Fusion of ilia to synsacrum: unfused (0); partially fused (1); well-fused (2). (K149) (K: fig.13; KC: fig.29) **Ordered**

204. Pelvis, preacetabular ilia: approach one another, but do not contact at midline (0); contact at midline forming canalis iliosynsacralis (1). **NOTE**: This character defines three states in KC181, KT187 and KF187; however, state 0 does not appear in any of the included taxa. Consequently, we keep only states 1 and 2 described in those works, recoding them as 0 and 1 respectively. (KC181)

205. Pelvis, foramina intertransversalia large, forming wide openings on dorsal surface of pelvis: absent (0); present (1). (KC182)

206. Ilium, projected postiliac spine: absent (0); present (1).

207. Pelvis, size of foramen ilioischiadicum and foramen acetabuli: foramen ilioischiadicum smaller or similar in size (0); larger (1). (OH16) (BG: fig.24; KC: fig.29)

208. Pelvis, fenestra ischiopubica: very wide and closed at its caudal end (0); slit-like and open at its caudal end (1). (BG133)

209. Ischium, most caudal extent in relation to postacetabular ilium: ischium shorter than ilium (0); ischium projects slightly beyond the ilium (1); ischium produced far caudal to ilium (2). (BG134) (BG: fig.24)

210. Patella: absent or unossified (0); present (1). (KC187)

211. Patella, sulcus m. ambiens: shallow groove (0); deep groove (1); perforated (2). (BG135) (OH: fig.8; BG: fig.25)

212. Tibiotarsus, crista patellaris: slightly developed (0); moderate enlarged (1); greatly enlarged (2). (BG136) **NOTE**: In KC189, KF196 and KF195 only two states are defined. However, the matrices include three states. These are the three states used in their matrices.

213. Tibiotarsus, shaft, anteroposterior flattening: weak, midshaft anteroposterior depth greater than 75% mediolateral width (0); strong, midshaft anteroposterior depth equal to or less than 75% mediolateral width (1). (C169)

214. Tibiotarsus, notch in distal edge of medial condyle (condylus medialis): present (0); absent (1). (AH38)

215. Tibiotarsus, lateral condyle (condylus lateralis) in lateral profile: ovoid (0); subcircular (1). (AH37)

216. Tibiotarsus, sulcus extensorius: laterally positioned (0); close to midline (1); medially positioned (2). **NOTE**: Variation of this feature in penguins was noted by Clarke et al. (2003). (KC193).

217. Tibiotarsus, medial margin in distal view: margin is nearly straight (0); margin strongly convex (1). (KF242)

218. Tarsometatarsus, elongation index (proximodistal length / mediolateral width at proximal end): elongated, EI ≥ 3 (0); slender, 3 > EI ≥ 2.5 (1); shortened, 2.5 > EI ≥ 2 (2); greatly shortened, EI < 2 (3). **NEW STATE**: State 2 can be seen in *Palaeospheniscus* and *Eudyptula*; whereas the new state 3 can be seen in *Nucleornis* and

Aptenodytes. This latter state was included in state 2 of K156, KC194, KT200 and KF200. Values for some Antarctic fossils were obtained from the table of measurements in Myrcha et al. (2002). (BG138) **Ordered**

219. Tarsometatarsus, collateral lateral ligament scar (impressio lig. collat. lat.): absent or inconspicuous (0); well defined creating a depression over the lateral surface (1); well-defined creating a notch on the proximolateral vertex (2). **NEW CHARACTER**: State 0 can be seen in *Eudyptes*, state 1 in *Pygoscelis* and state 2 in *Spheniscus*.

220. Tarsometatarsus, medial margin, pronounced convexity: absent (0), present (1). (K157)

221. Tarsometatarsus, enclosed hypotarsal canals (canales hypotarsi): present (0); absent (1). (BG141)

222. Tarsometatarsus, relative plantar projection of medial and lateral hypotarsal crests: medial crest projects farther than lateral (0); projection of medial and lateral hypotarsal crests subequal (1). (KT203)

223. Tarsometatarsus, intermediate hypotarsal crests (crista intermediae hypotarsi): distinct and well defined in plantar view, separated by groove (0); united with lateral crest, slightly separated by shallow groove in proximal view (1); indistinguishable or absent (2). **NEW STATE**: Under this new definition, state 0 can be seen in *Mesetaornis*, the new state 1 in *Palaeospheniscus* and state 2 in *Spheniscus*. (K158) (K: fig.14-15)

224. Tarsometatarsus, lateral hypotarsal crest (crista lateralis hypotarsi): enlarged and connected with medial crest (0); well defined and parallel to proximodistal axis of tarsometatarsus (1); reduced, poorly defined and proximal to lateral foramen (2); forming a diagonal ridge that overhangs lateral foramen (3). **NEW CHARACTER**: State 0 can be

seen in *Gavia*, state 1 in *Thalassarche*, state 2 in *Pygoscelis* and state 3 in *Palaeospheniscus*.

225. Tarsometatarsus, crista medialis hypotarsi: present (0); absent (1). (KF243) (KF: fig.7l,p,r)

226. Tarsometatarsus, dorsal sulcus between metatarsals II and III (sulcus longitudialis dorsalis medialis): absent or barely perceptible (0); shallow groove (1); moderate groove (2) deep groove (3). (K159) (K: fig.15) **Ordered**

227. Tarsometatarsus, proximal vascular foramina on plantar surface: foramen vasculare proximale mediale present, foramen vasculare proximale laterale absent or vestigial (0); both foramina present (1); foramen vasculare proximale laterale present, foramen vasculare proximale mediale absent or vestigial (2). **NOTE**: State 1 refers to the plantar opening of the medial foramen; regardless if it is open at the plantar surface as in *Pygoscelis*, or at the medial surface of the medial hypotarsal crest as in *Spheniscus*. (K162) (K: fig.14-15; KC: fig.30)

228. Tarsometatarsus, medial hypotarsal crest (crista medialis hypotarsi) perforated by opening for the medial foramen proximalis vascularis: absent (0); present (1). (BG139) (BG: fig.26)

229. Tarsometatarsus, proximal vascular foramen lateral on dorsal surface: absent or vestigial (0); small (1); enlarged (2). **NEW CHARACTER**: Although there is a large quantitative variation in the size of the vascular foramina, the extreme morphologies described for states 0 and 2 are exclusive of some taxa. State 0 can be seen in *Eretiscus*, state 1 in *Eudyptes* and state 2 in *Spheniscus humboldti*.

230. Tarsometatarsus, opening for medial foramen proximalis vascularis distal to crista medialis hypotarsi: absent (0); present (1). **NOTE**: Because both this opening and an additional opening perforating the crista medialis hypotarsi can be present in *Aptenodytes,* they are treated as independent characters. (BG140)

231. Tarsometatarsus, distal vascular foramen (foramen vasculare distale): present, separated from incisura intertrochlearis lateralis by osseous bridge (0); present, open distally (1); absent (2). (K163) (K: fig.15; KC: fig.30) **Ordered**

232. Tarsometatarsus, os metatarsale IV: distal end projects laterally (0); straight (1), distal end deflected medially (2). (K 160)

233. Tarsometatarsus, intertrochlear notches (incisura intertroclear): medial notch absent (0); medial notch deeper than lateral (1); sub-equal to equal deepness (2); lateral notch deeper than medial (3). **NEW CHARACTER**: This character is clearly visible in plantar view. State 0 can be seen in *Gavia,* state 1 in *Puffinus*, state 2 in *Aptenodytes* and state 3 in *Eudyptes*.

234. Tarsometatarsus, trochleae metatarsi II and IV in dorsal view: trochlea II shorter than IV (0); trochlea IV slightly shorter than II (1); equal (2). **NEW CHARACTER**: State 0 can be seen in *Gavia*, state 1 in *Eudyptes* and state 2 in *Spheniscus.*

235. Tarsometatarsus, trochleae in distal view: trochleae metatarsi III and IV aligned in same plane (0); trochlea metatarsi IV displaced dorsally (1). (KT211) (KT: fig.1w-y)

236. Tarsometatarsus, trochlea metatarsi II strongly plantarly deflected in distal view: no (0); yes (1). **NOTE**: This character refers to the plantar edge of the trochlea II with respect to the plane defined by the most plantar point of the trochleae III and IV in distal view; or with respect to the plane defined by the trochlear ridges of the trochlea III when

the trochlea IV is strongly dorsally deflected. State 0 can be seen in *Eudyptes* and state 1 in *Palaeospheniscus*. (A73)

237. Pedal digit I: small, with metatarsal I and single phalanx both present (0); metatarsal I reduced to an ossicle, claw represented by a minute ossicle or lost (1); metatarsal I absent (2).**NOTE**: Codings for Procellariiformes follow Forbes (1882); see also discussion in Mayr (2009). (KF245) **Ordered**

Myology

238. M. latissimus dorsi, pars cranialis, accessory slip: absent (0); present (1). (BG143)

239. M. latissimus dorsi, pars cranialis and pars caudalis: separated (0); fused (1). (BG144)

240. M. latissimus dorsi, pars metapatagialis, development: wide (0); intermediate (1); narrow (2). (BG145) **Ordered**

241. M. serratus profundus, cranial fascicle: absent (0); present (1). (BG146)

242. M. deltoideus, pars propatagialis, subdivision in superficial and deep layers: undivided (0); divided (1). (BG147)

243. M. deltoideus, pars major: triangular or fan-shaped (0); strap-shaped (1). (BG148)

244. M. deltoideus, pars major, caput caudale: short (0); intermediate (1); long (2). (BG149) **Ordered**

245. M. deltoideus, pars minor, origin on the clavicular articulation of the coracoid: absent (0); present (1). (BG150)

246. M. ulnometacarpalis ventralis: absent (0); present (1). (BG151)

247. M. iliotrochantericus caudalis: narrow (0); wide (1). (BG152)

248. M. iliofemoralis, origin: tendinous (0); partially tendinous and partially fleshy (1); totally fleshy (2). **NOTE:** This character previously included four states. The states 'mostly tendinous' and 'mostly fleshy' were lumped into a single state to avoid overweighing this ordered character. (BG153) **Ordered**

249. M. flexor perforatus digitis IV, rami II-III: free (0); fused (1). (BG154)

250. M. flexor perforatus digitis IV, rami I-IV: free (0); fused (1). (BG155)

251. M. flexor perforatus digitis IV, insertion of middle rami: on phalanx 3 (0); on phalanx 4 (1). (BG156)

252. M. latissimus dorsi, pars caudalis, additional origin from dorsal process of vertebrae: absent (0); present (1). (BG157)

Other soft tissue

253. Oral mucosa (bucca, tunica mucosa oris), buccal papillae group on the medial surface of the lower jaw (ramus mandibularis) at the level of the rictus: small number of rudimentary papillae with no clear arrangement (0); two clear rows of short conical papillae (1); large, elongated papillae with no clear arrangement (2). (BG158)

254. Tracheal rings: single (0); bifurcated (1). (KC219)

Consensus trees

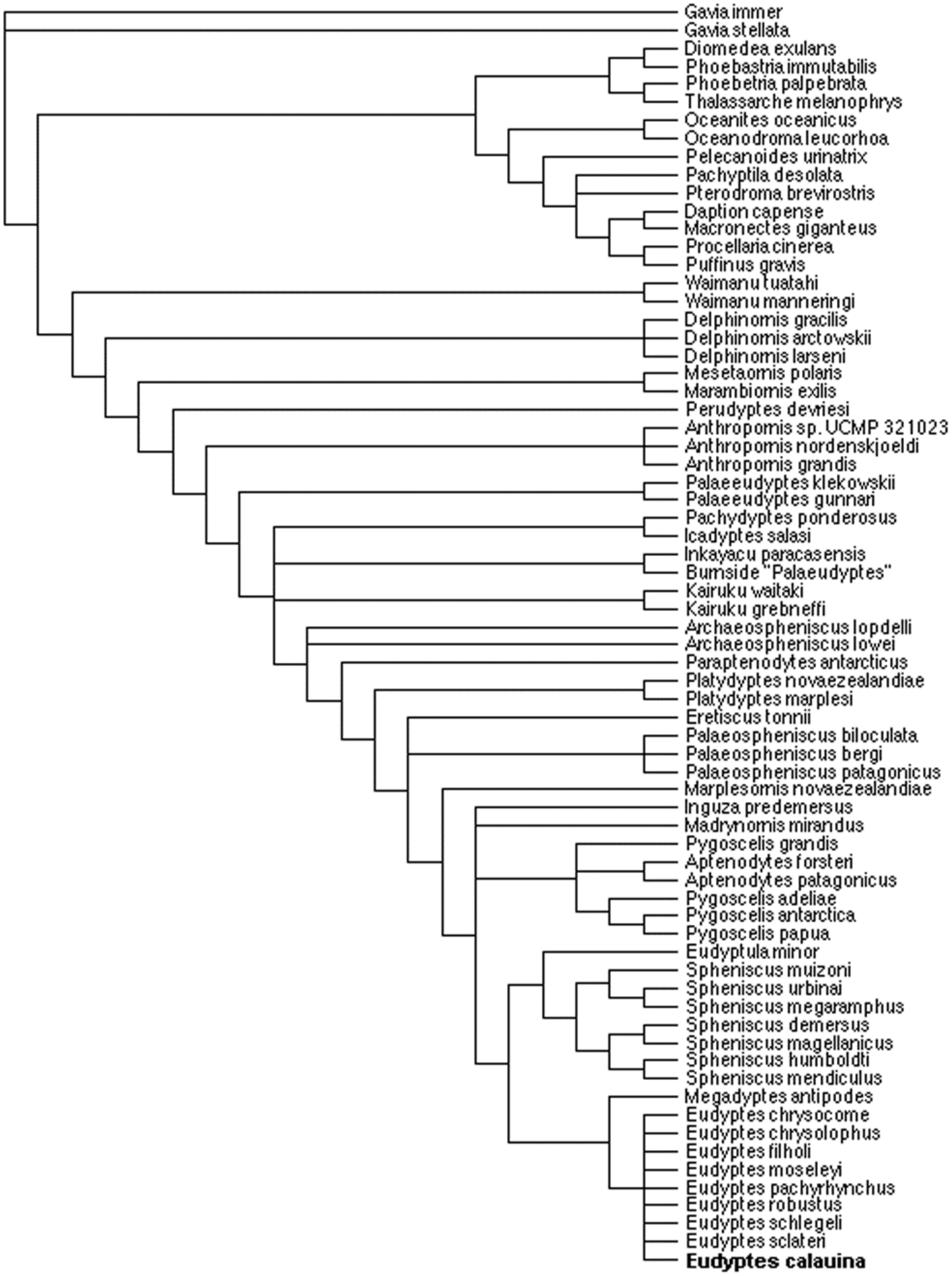

Figure S3.1. Combined analysis strict consensus. Strict consensus tree of 192 MPTs (tree length = 5563 steps, rescalated consistency index [RC] = 0.373, retention index [RI] = 0.699) from a combined analysis of morphological characters plus >6000 bp.

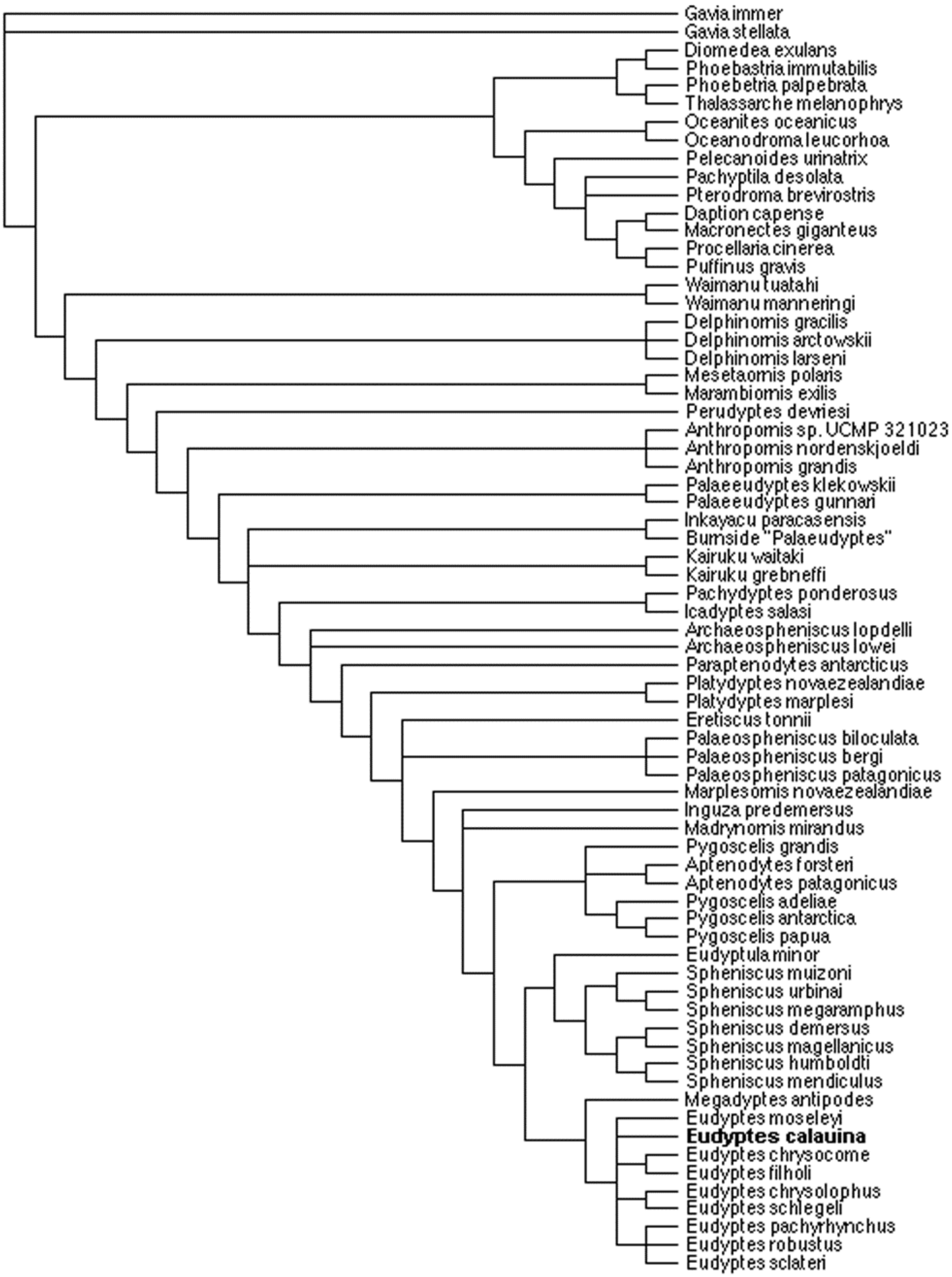

Figure S3.2. Combined analysis Adams consensus. Adams consensus tree of 192 MPTs (tree length = 5563 steps, RC = 0.373, RI = 0.699) from a combined analysis of morphological characters plus >6000 bp.

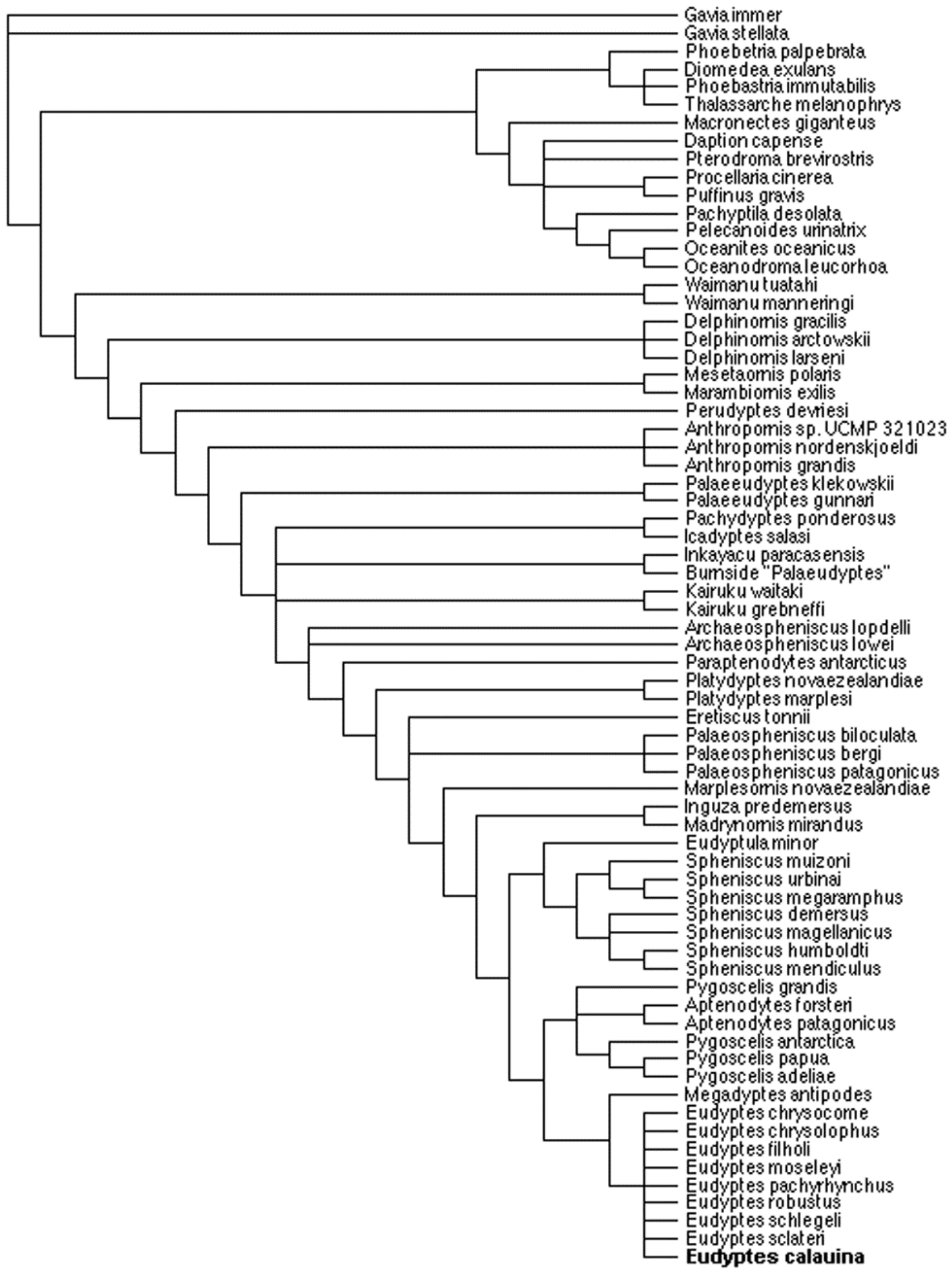

Figure S3.3. Morphology-only analysis strict consensus. Strict consensus tree of 704 MPTs (tree length = 802 steps, RC = 0.492, RI = 0.879) from an analysis of 254 morphological-only characters.

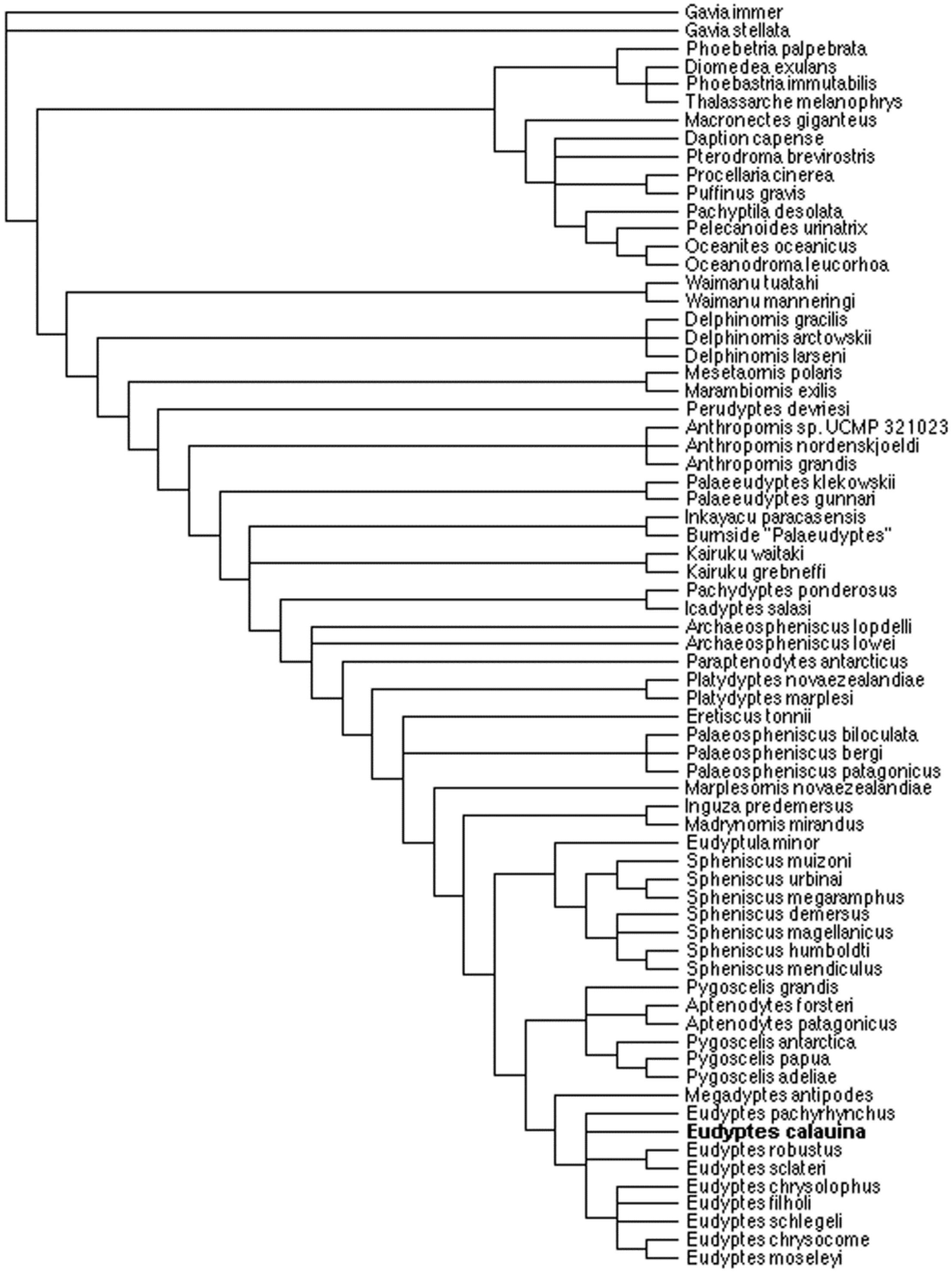

Figure S3.4. Morphology-only analysis Adams consensus. Adams consensus tree of 704 MPTs (tree length = 802 steps, RC = 0.492, RI = 0.879) from an analysis of 254 morphological-only characters.

List of osteological synapomorphies

Osteological synapomorphies (unambiguous and supportive) for main clades and genera monophyly, obtained from the combined analysis.

Clade/Taxa	Unambiguous synapomorphies	Supportive synapomorphies
Mesetaornis + *Marambiornis*	218(0)	—
Palaeeudyptes	—	157(0); 189(1)
Inkayacu + Burnside "*Palaeeudyptes*"	169(0); 171(1)	77(0); 140(1); 180(0); 236(1)
Pachydyptes + *Icadyptes*	177(0)	109(1); 157(0)
Palaeospheniscus	223(1); 236(1)	126(1); 127(0); 133(2); 163(1); 180(1)
Spheniscidae + *Madrynornis* + *Inguza*	88(0); 99(0); 106(1); 116(1); 119(2); 137(1)	98(2); 110(1); 118(1)
Spheniscidae excluding *Madrynornis* and *Inguza*	103(1); 136(0); 147(1); 224(2)	77(0); 105(1); 103(1); 158(2); 177(1)
Antarctic clade	83(0); 89(1); 90(1); 148(1); 151(1); 218(3); 219(1); 230(1)	102(3); 118(0); 211(0); 228(0); 233(2)
Aptenodytes	92(1); 111(1); 126(1); 133(0); 186(0); 219(0)	101(1); 120(1); 158(1)
Pygoscelis	84(1); 87(1); 115(1); 119(1); 203(1); 222(1)	160(0); 211(2)
Temperate-Tropical clade	226(2)	82(0); 180(1)
Burrowing clade	99(1); 102(1); 129(2); 156(1); 157(1); 226(3)	105(2); 207(1)
Eudyptula	125(3); 148(1); 176(2); 236(1)	177(0)
Pan-*Spheniscus*	78(1); 79(1); 80(1); 88(1); 110(0); 126(1); 163(1)	84(0)
Stem *Spheniscus*	136(1); 147(0)	207(0)
Crown *Spheniscus*	115(1); 118(2); 162(1); 235(1)	—
Yellow-headed clade	87(1); 91(0); 110(2); 115(1); 219(0)	112(1)
Megadyptes	—	233(2)
Eudyptes	102(3); 113(1); 218(3); 224(3); 234(1)	105(2); 177(0)

Additional References

Acosta Hospitaleche C, Gasparini G (2007) Evaluation for systematic purposes of the tarsometatarsal characters in Spheniscidae. Ornitologia Neotropical 18: 277-284.

Acosta Hospitaleche C, Tambussi C, Donato M, Cozzuol M (2007) A new Miocene penguin from Patagonia and its phylogenetic relationships. Acta Palaeontologica Polonica 52: 299- 314.

Baker AJ, Pereira SL, Haddrath OP, Edge A (2006) Multiple gene evidence for expansion of extant penguins out of Antarctica due to global cooling. Proceedings of the Royal Society B 217: 11–17

Banks J, Van Buren A, Cherel Y, Whitfield JB (2006) Genetic evidence for three species of rockhopper penguins, *Eudyptes chrysocome*. Polar Biology 30: 61-67.

Bertelli S, Giannini N P (2005) A phylogeny of extant penguins (Aves: Sphenisciformes) combining morphology and mitochondrial sequences. Cladistics 21: 209-239.

Clarke JA, Ksepka DT, Salas-Gismondi R, Altamirano AJ, Shawkey MD, *et al*. (2010) Fossil evidence for evolution of the shape and color of penguin feathers. Science 330: 954-957.

Clarke JA, Ksepka DT, Stucchi M, Urbina M, Giannini N, *et al*. (2007) Paleogene equatorial penguins challenge the proposed relationship between biogeography, diversity, and Cenozoic climate change. Proceedings of the National Academy of Sciences of the United States of America 104**:** 11545-11550.

Cooper A, Penny D (1997) Mass Survival of Birds across the Cretaceous- Tertiary Boundary: Molecular Evidence. Science 275: 1109–1113.

Ericson PGP, Anderson CL, Britton T, Elzanowski A, Johansson US, *et al.* (2006) Diversification of Neoaves: integration of molecular sequence data and fossils. Biology latters 2: 543-547.

Giannini NP, Bertelli S (2004) Phylogeny of extant penguins based on integumentary and breeding characters. Auk 121: 422-434.

Göhlich U (2007) The oldest fossil record of the extant penguin genus *Spheniscus*, a new species from the Miocene of Peru. Acta Palaeontologica Polonica 52: 285–298.

Guinard G, Marchand D, Courant F, Gauthier-Clerc M, Le Bohec C (2010) Morphology, ontogenesis and mechanics of cervical vertebrae in four species of penguins (Aves: Spheniscidae). Polar Biology 33: 807-822.

Hebert PDN, Stoeckle MY, Zemlak TS, Francis CM (2004) Identification of Birds through DNA Barcodes. PLoS Biol 2: e312.

Kerr KCR, Stoeckle MY, Dove CJ, Weigt LA, Francis CM, *et al*. (2007) Comprehensive DNA barcode coverage of North American birds. Molecular Ecology Notes 7: 535–543.

Ksepka DT, Bertelli S, Giannini NP (2006) The phylogeny of the living and fossil Sphenisciformes (penguins). Cladistics 22: 412-441.

Nunn GB, Stanley SE (1998) Body size effects and rates of cytochrome b evolution in tube-nosed seabirds. Molecular Biology and Evolution 15: 1360-1371.

Nunn GB, Cooper J, Jouventin P, Robertson CJR, Robertson GG (1996) Evolutionary relationships among extant albatrosses (Procellariiformes: Diomedeidae) established from complete cytochrome-b gene sequences. Auk 113: 784-801.

Paterson AM, Wallis GP, Gray RD (1995) Penguins, petrels, and parsimony: does cladistic analysis of behavior reflect seabird phylogeny? Evolution 49: 974-989.

Slack KE, Jones CM, Ando T, Harrison GL, Fordyce RE, *et al*. (2006) Early Penguin Fossils, plus Mitochondrial Genomes, Calibrate Avian Evolution. Molecular Biology and Evolution 23: 1144-1155.

Stanley SE, Harrison RG (1999) Cytochrome b evolution in birds and mammals: an evaluation of the avian constraint hypothesis. Molecular Biology and Evolution 16: 1575-1585.

Stephan B (1979) Vergleichende Osteologie der Pinguine. Mitteilung aus dem Zoologischen Museum in Berlin 55: 3- 98.

Van Tuinen M, Sibley CG, Hedges SB (2000) The Early History of Modern Birds inferred from DNA Sequences of Nuclear and Mitochondrial Ribosomal Genes. Molecular Biology and Evolution 17: 451-457.

Worthy TH (1997) The identification of fossil *Eudyptes* and *Megadyptes* bones at Marfells Beach, Marlborough, South Island. New Zealand Natural Sciences 23: 71-85.
